# MetAP-like Ebp1 occupies the human ribosomal tunnel exit and recruits flexible rRNA expansion segments

**DOI:** 10.1038/s41467-020-14603-7

**Published:** 2020-02-07

**Authors:** Klemens Wild, Milan Aleksić, Karine Lapouge, Keven D. Juaire, Dirk Flemming, Stefan Pfeffer, Irmgard Sinning

**Affiliations:** 1 Biochemiezentrum der Universität Heidelberg (BZH), INF 328, D-69120 Heidelberg, Germany; 2 Zentrum für Molekulare Biologie der Universität Heidelberg, INF282, D-69120 Heidelberg, Germany

**Keywords:** RNA, Ribosome, Cryoelectron microscopy

## Abstract

Human Ebp1 is a member of the proliferation-associated 2G4 (PA2G4) family and plays an important role in cancer regulation. Ebp1 shares the methionine aminopeptidase (MetAP) fold and binds to mature 80S ribosomes for translational control. Here, we present a cryo-EM single particle analysis reconstruction of Ebp1 bound to non-translating human 80S ribosomes at a resolution range from 3.3 to ~8 Å. Ebp1 blocks the tunnel exit with major interactions to the general uL23/uL29 docking site for nascent chain-associated factors complemented by eukaryote-specific eL19 and rRNA helix H59. H59 is defined as dynamic adaptor undergoing significant remodeling upon Ebp1 binding. Ebp1 recruits rRNA expansion segment ES27L to the tunnel exit via specific interactions with rRNA consensus sequences. The Ebp1-ribosome complex serves as a template for MetAP binding and provides insights into the structural principles for spatial coordination of co-translational events and molecular triage at the ribosomal tunnel exit.

## Abstract (web summary)

The ErbB3 receptor binding protein Ebp1 binds to ribosomes and is linked to translational control. Here, the authors present the cryo-EM structure of human Ebp1 bound to a non-translating 80S ribosome and find that Ebp1 blocks the tunnel exit and recruits the rRNA expansion segment ES27L to the tunnel exit.

## Introduction

The ErbB3 receptor-binding protein (Ebp1, 394 residues) is a highly conserved, widely expressed, and multifunctional eukaryotic protein. It has attracted considerable attention due to its regulative role in cancer progression, although the question of being “friend or foe”^1^ is still unanswered. This uncertainty is based on the contradictory functions of the two splice variants p42 and p48, with p42 (lacking 54 N-terminal residues) acting as tumor suppressor and p48 promoting cell proliferation^1^. First described as part of the ErbB3 receptor pathway^2^, it has been further on identified as an IRES-*trans*-acting factor (ITAF45) initiating viral mRNA translation^3^. Moreover, Ebp1 binds to ribosomes and was also shown to inhibit phosphorylation of initiation factor eIF2α^4^. Ebp1 itself is phosphorylated on multiple Ser/Thr sites, which promotes interactions with ErbB3 and Akt kinase involved in apoptosis (Ser360 phosphorylation) (reviewed in ref. ^1^). Further kinases described to interact with Ebp1 are protein kinase C (PKCδ), dsRNA-activated kinase (PKR), p21-activated serine–threonine kinase (PAK1), and cyclin-dependent kinase (CDK2).

X-ray structures of Ebp1^5,6^ confirmed the predicted methionine aminopeptidase (MetAP) fold, also described as pita-bread fold, with a deep pocket on its concave surface accommodating the active site in MetAPs^7^. MetAPs are metalloproteases essential for all kingdoms of life that cleave-off the first methionine from the growing polypeptide chain as soon as a length of 40 amino acids is reached^8^. Together with the ribosome-associated chaperones RAC and Ssb, they belong to the first interaction partners of nascent chains (NCs) emerging from the ribosome^9,10^. MetAPs are classified into two types^7^, with MetAP-2 including a helical subdomain (insert domain) of ~60 residues (Supplementary Fig. 1). Ebp1 is MetAP-2 like and mainly distinguished from the MetAPs by the missing catalytic activity, a shorter N-terminus (~150 residues missing) and a C-terminal extension of about 50 residues harboring a highly positive charged patch including six consecutive lysine residues (Supplementary Fig. 1). The lysine cluster has been shown to act as major nucleolar localization signal and to be involved in phosphoinositide binding^11^. Positive surface charges of Ebp1, indicative for the confirmed dsRNA-binding properties of Ebp1^5^, cluster on the convex side and include a charged surface loop (residues 62–72) and the C-terminal region, although the lysine cluster was disordered and not part of the X-ray models (missing 30 C-terminal residues)^5,6^.

Ebp1 has been further on assigned as the human homolog of the 60S pre-ribosomal nuclear export factor Arx1 from *Saccharomyces cerevisiae*^12^, but any direct evidence for such functional homology is still missing. In particular, Ebp1 lacks structural features that are centrally involved in ribosome biogenesis factor interaction in yeast Arx1^13,14^, and it does not bind to nucleoporins^12^, as would be required for a pre-60S nuclear export factor. Furthermore, Ebp1 stably binds to mature 80S ribosomes in vivo as shown in this study, while Arx1 is known to exclusively bind to 60S ribosomal subunits during maturation. Intermediate resolution cryo-electron microscopy (cryo-EM) structures of Arx1 bound to the yeast 60S (pre-)ribosome were reported previously^13,15^, and finally refined to 3.4 Å resolution^14^. These cryo-EM reconstructions confirmed the structural homology to the MetAPs and visualized Arx1 in the same binding site on the ribosomal tunnel exit that was observed for a bacterial MetAP–ribosome complex in a low-resolution structure of >10 Å^16^.

Recently, MetAPs enzymatic activity was shown in yeast to depend on its interaction with rRNA expansion segment ES27L, one of the longest tentacle-like dsRNA insertions (714 nucleotides in human 28S rRNA) typical for eukaryotic ribosomes^17^. Unexpectedly, this interaction was at the same time found to control the accuracy of ribosomal mRNA decoding. The mechanism is elusive, and sequence conservation in ESs is generally low and varies within species and even tissues^18^. Deletion of ES27L is lethal in the ciliate *Tetrahymena thermophila*^19^, and its close proximity to the tunnel exit perfectly positions it for the coordination of co-translational interaction partners. The exact role of ESs in translational control, the precision of protein biosynthesis, and maturation and folding is not understood.

To shed light on the role of Ebp1 in translational regulation and molecular mechanisms underlying its multiple cellular functions, we wanted to address its structure in the context of the translation machinery using cryo-EM single-particle analysis. Here, we present the cryo-EM reconstruction of Ebp1 bound to the human 80S ribosome at a resolution ranging from 3.3 to ~8 Å. Ebp1 binds on top of the tunnel exit by several distinct interaction sites, with a remodeled rRNA helix H59 and ES27L presenting the most prominent contacts. Our data provide a rationale of the structure, dynamics, and function of ESs at the ribosomal tunnel exit. The structure provides a generalized view of MetAP-fold recognition by the ribosome, and reveals structural principles for spatial coordination of co-translational events at the ribosomal tunnel exit.

## Results

### Cryo-EM structure of Ebp1 bound to the human 80S ribosome

**Fig. 1:**
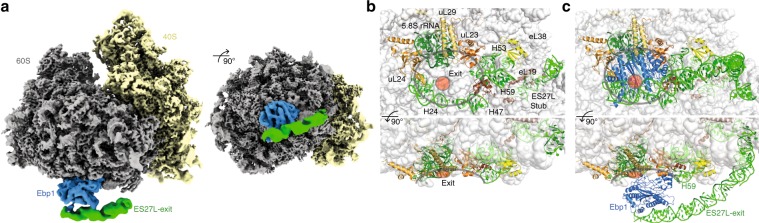
Ebp1 binds to the ribosomal tunnel exit and ES27L. **a** Composite cryo-EM reconstruction of the Ebp1–ribosome complex after 3-body refinement. The ribosomal large (gray) and small (yellow) subunits, Ebp1 (blue) and ES27L (green) are depicted. **b**, **c** Overall structures of the vacant human ribosome (PDB ID: 6EK0) and the Ebp1–ribosome complex. Cartoon representations for ribosomal elements involved in Ebp1 binding (rRNA: green; ribosomal proteins: yellow, orange, brown) are shown for the vacant ribosome (**b**) and in complex with Ebp1 (blue, **c**). Ribosome components not engaged in Ebp1 interaction are shown in a transparent surface representation. ES27L (green) is only visible as stub without any associated factor and is fixed in the exit position upon Ebp1 binding. The ribosomal tunnel exit (red circle) and the inner ring of ribosomal components around the tunnel exit (gray outline) are indicated. Views are the same as in (**a**) with panels in the upper row looking straight down onto the ribosomal tunnel exit, while panels in the lower row are rotated by 90°.

Binding of factors to the ribosomal surface is often governed by considerable flexibility and conformational heterogeneity that can only be addressed by a hybrid structural biology approach integrating state-of-the-art cryo-EM analysis and high-resolution X-ray structures. The 1.6 Å X-ray structure of Ebp1 was solved in our lab previously^5^. To gain insights into Ebp1 binding to the human 80S ribosome, we used cryo-EM single-particle analysis, and determined the structure of full-length Ebp1 (p48 isoform) in complex with puromycin-treated 80S ribosomes purified from HeLa cells (Supplementary Fig. 2, Supplementary Table 1). Interestingly, ~25% of the purified ribosomes were already decorated with endogenous Ebp1 (dataset 1), and this fraction could be raised up to ~75% upon addition of recombinant Ebp1 (dataset 2). The in vivo pulled-out and in vitro-reconstituted Ebp1–ribosome complexes were virtually identical, indicating that no significant conformational changes or rearrangements were introduced by reconstitution of the complex with the purified ribosomes. Therefore, the two datasets were merged to obtain a single higher resolution structure (Fig. 1a). In brief, ribosomal particles were automatically located and subjected to several consecutive rounds of in silico sorting. Initial refinement of the 34,467 particles retained after sorting resulted in a reconstruction at 3.3 Å global resolution (Supplementary Fig. 3a). Lower local resolution for the 40S ribosomal subunit (Supplementary Fig. 3d) indicated conformational heterogeneity originating from ribosomal intersubunit rotation, which we compensated for by separating the Ebp1–ribosome complex into two independently refined segments (“2-body” approach), comprising the 40S ribosomal subunit and the 60S ribosomal subunit plus Ebp1 and rRNA ES27L, respectively. This approach resulted in improved global and local resolution (Supplementary Fig. 3b, e) and density quality for the two ribosomal subunits and directly ribosome-associated Ebp1 segments. This set of cryo-EM densities thus allowed us to analyze the interface between Ebp1 and the ribosomal tunnel exit at a resolution of better than 4 Å (Supplementary Fig. 3e, g), with amino acid side chains and rRNA bases clearly resolved for the entire interface. In contrast, lower local resolution for more peripheral regions of Ebp1 and ES27L hindered interpretation of the Ebp1–ES27L interface, and suggested additional conformational mobility independent from the ribosomal 60S subunit for these two components (Supplementary Fig. 3b). We thus subjected particles to another round of refinement, in which we treated the Ebp1–ES27L segment as a separately refined body (“3-body” approach). Local resolution and interpretable features for ES27L and the very peripheral Ebp1 segments interacting with ES27L improved using this approach (Supplementary Fig. 3c, f, h), and the resulting cryo-EM density thus allowed detailed analysis of the Ebp1–ES27L interface. Supplementary Fig. 4 shows the full set of cryo-EM densities obtained from the three individual refinement approaches and highlights the density segments that were used to address the different aspects of the Ebp1–ribosome interaction.

### Ebp1 occupies the ribosomal tunnel exit and recruits ES27L

The MetAP fold of Ebp1 as determined by X-ray structure analysis binds to the 80S ribosome as a rigid body (root-mean-square deviation (rmsd) for 352 Cα-atoms of 1.0 Å) and thus the model could be directly fitted into the cryo-EM density (real-space correlation coefficient (cc) of 64%). In particular, the relative orientation of the insert domain in respect to the protein core remains unchanged. Ebp1 binds with its concave surface, harboring the active site in MetAPs, directly on top of the ribosomal tunnel exit and is sandwiched between the exit and ES27L (Fig. 1a). Thus, access to the tunnel exit is completely blocked for any globular NC-binding factor, and only a small lateral gap between Ebp1 and the tunnel exit is left (Supplementary Fig. 5). Ebp1 establishes contacts with ribosomal components all around the inner ring of the tunnel exit, including proteins eL19, uL23, uL24, and uL29 as well as the 5.8S rRNA (helix 24) and the 28S rRNA (helices H24, H47, H53, and H59) (Fig. 1b, c). The conformations of all these ribosomal elements are almost identical as previously described in cryo-EM structures of the vacant human 80S ribosome^20–22^. The single and pronounced exception is rRNA helix H59, which undergoes significant remodeling upon Ebp1 binding, identifying it as a conformationally dynamic adaptor on the 60S ribosomal subunit.

Owing to their dynamic behavior, long ribosomal RNA expansion segments are typically not resolved in high-resolution cryo-EM reconstructions, with few exceptions in which their conformational landscape is confined by specific interactions with either the ribosomal core or ribosome-associated factors. For one of the longest eukaryotic rRNA ESs, ES27L, two distinct conformations were described^23,24^: ES27L either aligns with the ribosomal 40S/60S subunit interface (“L1-position”; “ES27L-L1”) or projects toward the ribosomal tunnel exit (“exit-position”; “ES27L-exit”) (Supplementary Fig. 6). The two conformations were suggested to play a role in coordinating access of non-ribosomal factors to the ribosomal tunnel exit^23^. In the human Ebp1–ribosome complex, ES27L is resolved in the exit position and the ES27L-B-arm (370 nucleotides, 2912–3281) reaches over the 60S tunnel exit (Fig. 1a, c) as previously only observed for 80S complexes from yeast^13,14,25^. The conformational changes within ES27L-B are immense, with an almost 120° rotation around its anchor point on the ribosomal surface (at ES27L-A of 28S rRNA) and maximal distances of the outermost visible parts (~1/3 of ES27L-B) moving more than 20 nm (Supplementary Fig. 6). The ES27L-B exit position is stabilized by an extensive contact of 1000 Å^2^ with Ebp1 (overall Ebp1–ribosome interaction surface: 2500 Å^2^) and an additional smaller contact with eL38. Even in the exit position, ES27L-B shows considerable plasticity in the eL38-binding region and is resolved throughout only after in silico particle sorting focused on the ES27L conformation (Supplementary Fig. 2d). Using this approach, several distinct ES27L-B conformations were identified with Ebp1 acting as a pivot accommodating various ES27L-B orientations (Supplementary Movie 1).

### The Ebp1–ES27L interaction

**Fig. 2:**
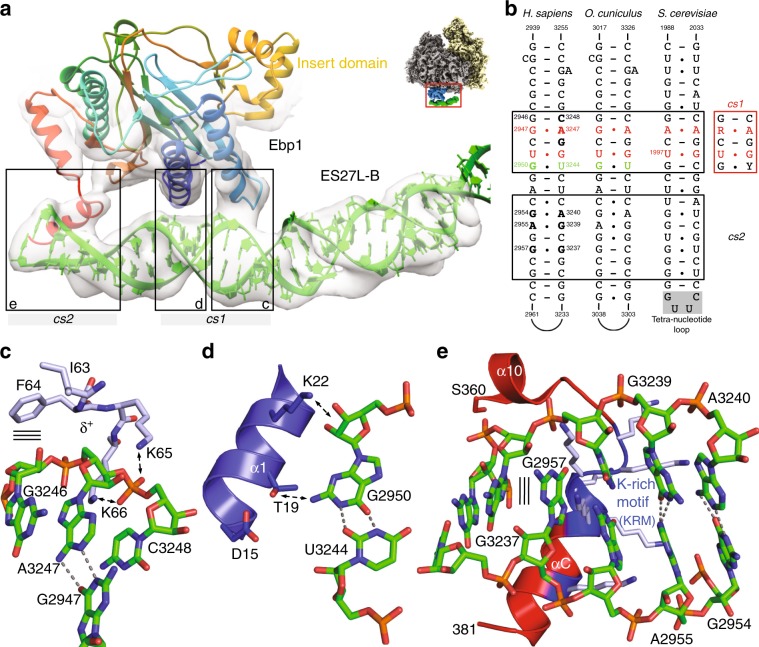
Conserved structural features of ES27L are instrumental in Ebp1 binding. **a** Three distinct interaction sites between Ebp1 and the consensus sequences *cs1* and *cs2* mediate ES27L binding. The atomic models for Ebp1 and ES27L are superposed to the cryo-EM density after 3-body multibody refinement. Density was faded out toward the Ebp1–ribosome contact, which is better resolved in the reconstruction from 2-body multibody refinement. View is the same as in Fig. 1a left panel and as indicated by the small representation in the corner. **b** Consensus sequences (*cs*) of ES27L involved in Ebp1 binding. Conserved mismatches within *cs1* are highlighted. **c**, **d** Structural details of ES27L interaction of the GA mismatch at *cs1* with the Ebp1 P-loop structure (δ^+^: partial positive charge) following helix α2 (**c**), and of the GU wobble with Ebp1 helix α1 (**d**). Putative protein–RNA interactions are indicated by arrows. **e** Interactions at *cs2* with the lysine-rich motif (KRM) within the Ebp1-specific C-terminal helix α C. The putative GG cross-strand purine stack is indicated by parallel lines.

MetAP binding to ES27L has been recently identified in yeast as an important principle increasing enzymatic activity and controlling translation fidelity^17^, but the underlying principles are unclear. ES27L sequences are generally not conserved in eukaryotes: they vary from an AU-rich base composition in *Drosophila* (32% GC) via an even content in yeast (57% GC) up to an extreme GC-rich version in *Homo sapiens* (89%)^20^ and ES27L length has been more than quadrupled from fungi (159 nts in baker’s yeast) to metazoans (714 nts in humans) for so far unknown reasons. Combining already available structural information from the Arx1–ES27L interaction in yeast with our cryo-EM reconstruction of the human Ebp1–ribosome complex, we could build a model for the corresponding regions of human ES27L, including 100 nts of ES27L-B reaching over the tunnel exit and parts of ES27L-C (30 nts). Although the base pairs are not resolved due to the extensive conformational plasticity of the central ES27L-B region (Supplementary Movie 1), the regular spacing of the A-form RNA helix emanating from the well-defined ES27L-A stem and resolved base pair mismatches allow for unambiguous extension of ES27L-B from the ribosomal core to Ebp1. The ES27L-B model allows for the definition of three specific Ebp1–ES27L contacts (Fig. 2a). Two of them involve N-terminal helices that are part of the conserved MetAP fold, while the last one is mediated by the Ebp1-specific C-terminal extension. On the RNA side, two consensus sequences are involved that are conserved from yeast to metazoans (Fig. 2b).

Consensus sequence 1 (*cs1*) contains two base mismatches, namely a purine–purine and a GU-wobble base pair, and is recognized by N-terminal regions of Ebp1 (Fig. 2a, b). The sterically demanding purine–purine interaction (G2947-A3247) pushes the phosphoribose backbone around A3247 into a positively charged loop (*K*^*62*^*IFKK*EKEMKK, interacting residues are in italics) on the convex side of Ebp1 (Fig. 2c). The “exposed” phosphate group of A3247 is accommodated in a “P-loop” (phosphate-binding loop)-like structure typical for the binding of the β-phosphate moieties of NTPs (N: any nucleotide) within the large superfamily of P-loop-containing NTPases (Pfam clan CL0023). In Ebp1, the phosphate approaches four main chain nitrogens oriented toward the phosphate group. The neighboring 5′-ribose of the conserved guanosine (G3246) is recognized by hydrophobic ring stacking on the two exposed hydrophobic Ebp1 residues Ile63 and Phe64, while the ribose of A3247 and the phosphate group of the 3′-nucleotide (C3248) are clamped in between the two conserved Ebp1 residues Lys65 and Lys66.

The GU-wobble base pair (G2950-U3244) within *cs1* is necessary in order to expose G2950 into the minor groove of the A-RNA helix where it is recognized by Thr19 on the N-terminal Ebp1 helix α1 (Fig. 2d). Interactions around the GU wobble are completed by Ebp1 residues exposed by neighboring turns of helix α1 (Asp15, Lys22). Both mismatch recognitions within *cs1* are conserved in yeast for the Arx1–ES27L interaction, as observed upon in-depth analysis of the original cryo-EM density^14^ by building the respective model for yeast ES27L (Supplementary Fig. 7a, b). However, ES27L in yeast has a slightly different orientation relative to Arx1 (rotational tilt), which results in an interaction of the neighboring GU wobble of *cs1* (U1997-G2024) with Arx1 (Fig. 2b; Supplementary Fig. 7b).

The adjacent consensus sequence 2 (*cs2*) is characterized by the accumulation of mismatches rather than sequence homology (Fig. 2b), which reflects the different readout principles mediated by the C-terminal Ebp1- and Arx1-specific regions. Human *cs2* is dominated by purine–purine mismatches, which overall result in a extensive widening of the major groove (by more than 75%) (Fig. 2a, e; Supplementary Fig. 7c) that typically is narrow and deep in A-RNA and not accessible for protein interactions. While the AG/GA tandem mismatch stretches the width of the RNA helix, it is constrained at the site of the GG mismatch (G2957/G3237) and best fitted by a cross-strand purine stack. The X-ray models of Ebp1^5,6^ lack the most C-terminal 33 amino acid residues directly following the important phosphorylation site Ser360 at the end of helix α10. In our cryo-EM reconstruction, the C-terminus of Ebp1 is resolved (Fig. 2a) and projects toward the widened major groove, where it forms an α helix (αC) with the highly basic lysine cluster (S^363^RKTQKK*KKKKASKTAENA*^381^, helical region in italics, side chains not traceable) that entirely fills the groove (Fig. 2e). This kind of major groove readout by positively charged α helices is well known from the so-called ARMs (arginine-rich motifs) as found i.e., in viral transactivation (HIV-1 Rev-peptide/Rev-response element RRE)^26^ and later on in the signal recognition particle (SRP68/SRP RNA)^27^. Accordingly, we define this motif as “KRM”, with the arginine (R) being replaced for lysine (K). In Arx1^14^, only one single turn of helix αC is formed, and the chain does not penetrate the major groove as the RNA continues in a regular A-form and yeast ES27L-B is terminated. The interaction of this single αC turn including two positive charges with the most distal ES27L-B GU wobble next to the tetranucleotide loop is reminiscent of the Ebp1–ES27L contact, but the overall architecture of the interaction site is different. All MetAPs are missing a corresponding positively charged C-terminal region (~50 residues less) and thus, this ES27L contact at *cs2* is specific for the PA2G4 family like Ebp1 and Arx1.

### The general docking site for NC-associated factors revisited

The second half of the Ebp1–ribosome interface is formed by the inner ring of the ribosomal tunnel exit. The tunnel exit is a hot spot in cellular activity and a plethora of NC-associated factors compete for similar binding sites (reviewed in refs. ^9,28^). Most importantly, uL23 has been described as a general docking site for NC-interacting chaperones (RAC, NAC, ERj1p, Trigger factor), protein targeting factors plus their associated membrane insertion machineries (SecA and SRP/translocon, Get pathway, Oxa1), and NC-modifying enzymes like the MetAPs.

**Fig. 3:**
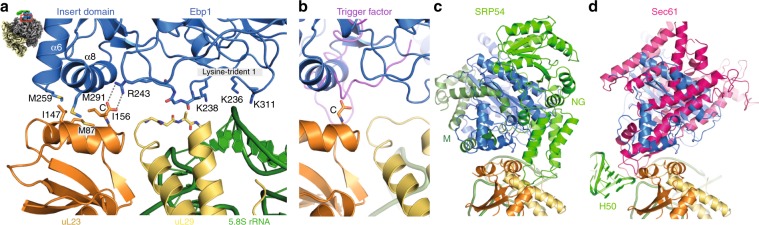
Ebp1 binding to the inner ring of the tunnel exit. **a** The insert domain of Ebp1 (blue) binds to the general docking site, including ribosomal proteins uL23 (yellow) and uL29 (orange). Three lysine residues within the MetAP-core fold triangulate 5.8S rRNA helix H24 (green). View is rotated in plane by 180° in respect to Fig. 1c bottom and as indicated by the small representation in the corner. **b** Bacterial Trigger factor^30^ (PDB ID: 1W2B) exploits the same binding mode to uL23. **c** The SRP GTPase SRP54 is incompatible with Ebp1/MetAP binding. The NG domain binds to uL29^32, 34^ (PDB ID: 3JAJ) and does not interfer with the insert domain, while signal-recognizing SRP54M (dark green) competes for uL23 binding (view rotated vertically by 90° to panel (**a**)). **d** The Sec61 translocon displaces SRP54 and reaches deep into the tunnel exit up to 28S rRNA H50^35, 36^ (PDB ID: 3J7Q).

In the Ebp1–ribosome complex, docking of the insert domain of Ebp1 to uL23 constitutes the largest interface to the inner tunnel exit ring (500 Å^2^) (Fig. 3a). The interface includes four α helices (α6 and α8 of Ebp1) and has a methionine-rich hydrophobic core that is surrounded by hydrophilic and charged contacts. Most strikingly, the very C-terminus of uL23 forms a salt bridge with Arg243 of Ebp1 at the start of the insert domain (Fig. 3a). The insert domain also contacts ribosomal elements around uL23, and thus this general docking site is rather extended (1200 Å^2^). Overall, the extended docking site confers to 80% of the Ebp1 interaction with the inner exit ring (1500 Å^2^). All minor interactions that complete the overall triangular docking of Ebp1 to the exit, form small “lysine-trident” contacts with either the phosphoribose backbone of 5.8S rRNA helix 24 (Fig. 3a), or with the connection of uL24/28S rRNA helix 24 (not shown).

This extended general docking site is unique among the so far described tunnel exit interactions. Still, the mode of central uL23 recognition is very similar as found for the bacterial Trigger factor^29^. The N-terminal domain of Trigger factor, a co-translational chaperone that crowches over the tunnel exit^30^, also forms hydrophobic interactions and binds the very C-terminus of bacterial uL23 (Fig. 3b). However, this contact constitutes the major interaction in the Trigger factor–ribosome complex, and binding of the chaperone to the exit is known to be transient^31^.

Also typical for many exit tunnel interactions, docking occurs in tandem with the uL23-neighboring protein uL29. In the Ebp1–ribosome complex, the contact with uL29 is small and appears rather flexible as it involves van der Waals interactions of small residues (glycines and alanines) (Fig. 3a). In comparison, SRP establishes a major interaction with both uL23 and uL29 when binding to the ribosome for targeting of nascent secretory and membrane proteins to the ER membrane^32–34^ (Fig. 3c). Interestingly, the insert domain of Ebp1 does not sterically exclude binding of the SRP GTPase SRP54, which is centered more toward uL29. However, the flexibly linked signal sequence-binding domain of SRP54 (SRP54M) cannot be positioned over the tunnel exit in the presence of Ebp1 (or MetAP-2). Similarly, the protein conducting channel of the ER membrane (Sec61αβγ) strongly binds to the same site^35,36^. While Sec61γ binds to uL23, Sec61α binds to uL29 and H50 of 28S rRNA at the base of the tunnel exit (Fig. 3d). Finally, cryo-EM reconstructions of the co-translational eukaryotic chaperones NAC (nascent polypeptide-associated complex)^37^ and the ER membrane protein ERj1p (Hsp40-type co-chaperone of ER-lumenal Hsp70 BiP)^38^ also revealed binding to the general docking site, however, due to limited resolution atomic models thereof could not be built and further data are necessary for detailed descriptions. The general physiological necessity for a triage between protein folding and co-translational targeting, depending on NC-associated factors binding to the general docking site, has been remarked and described earlier^9,28^.

### H59 forms a dynamic adaptor to the insert domain of Ebp1

**Fig. 4:**
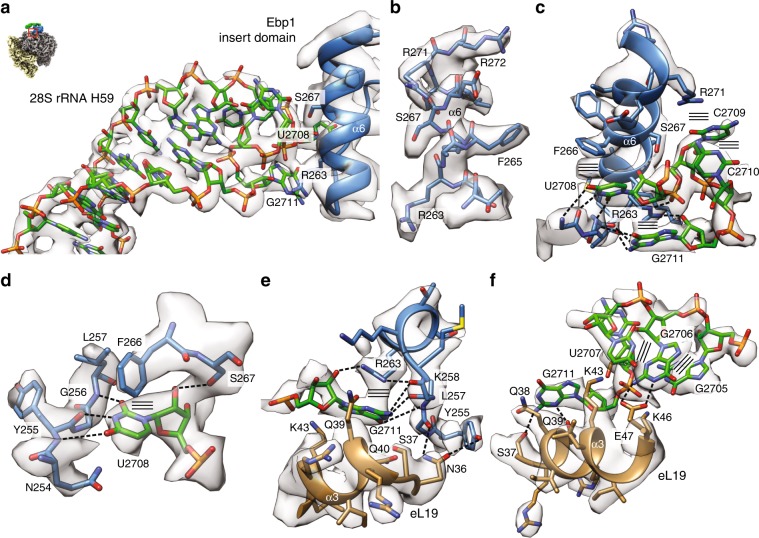
H59 forms a dynamic adaptor for Ebp1 binding. **a** The H59–Ebp1 interface. Atomic models of rRNA H59 (green) and Ebp1 helix α6 (blue) are superposed to the cryo-EM density (transparent gray). Overall orientation as in Fig. 3a and as indicated by the small representation in the corner. **b** Exemplary cryo-EM density of the Ebp1–ribosome interaction for the Ebp1 helix α6. **c** Structural details of the Ebp1–H59 interaction. Hydrogen bonds (dashed lines) and stacking interactions (parallel lines) are indicated. View is vertically rotated by 180° in respect to panels (**a**) and (**b**). **d** The Watson–Crick like readout of U2708. **e** Structural details of the Ebp1–H59–eL19 (brown) interaction around the bulged-out G2711 nucleotide. **f** H59 in the Ebp1-docked position rolled onto eL19 helix α3. In all panels, the cryo-EM density after 2-body multibody refinement is shown (transparent gray).

A striking feature of the Ebp1–ribosome interaction is the extension of the Ebp1-binding site beyond the general docking site toward eL19 and helix H59 of 28S rRNA (Fig. 4). This contact is even larger (700 Å^2^) than the uL23/uL29 interface, and unique among all other ribosomal complexes with available structural information (except of its yeast homolog Arx1^14^). Both eL19 and H59 (also termed ES24L; 21 nts in human 28S rRNA, 2698–2718) are eukaryote specific. In general, the globular N-terminal domain of eL19, which is essential for cell viability and ribosome biogenesis^39^, binds to H59 at its closing loop (G^2705^GUUCCG^2711^ in human 28S rRNA). In all mammalian ribosome structures solved so far, guanine G2711 is bulged out and anchors H59 on the ribosomal surface by interactions with H53 and the H58-59 connection (defined here as “locked position”) (Supplementary Fig. 8a). In the Ebp1–ribosome complex, the loop is completely remodeled and bridges over toward the highly basic helix α6 of the Ebp1 insert domain (Fig. 4a; Supplementary Fig. 8a, b). This remodeling coincides with a lateral displacement of H59 by up to 10 Å (at G2711), which releases the guanine anchor G2711 and rotates H59 further onto eL19 (“docked position”). Thus, H59 serves as a conformationally dynamic adaptor for Ebp1 recruitment to the tunnel exit (Supplementary Movie 2). It is unlikely that the observed remodeling of H59 also occurs in yeast, because the bulged-out nucleotide is not present and in all yeast ribosome structures H59 is found in the “Ebp1-like” docked position.

The cryo-EM density around the interaction site approximates the overall resolution of 3.3 Å, which allows for the precise fitting of the interacting partners and especially the region around Ebp1 helix α6 (Fig. 4b) as a main docking partner within the insert domain (Supplementary Fig. 1). Overall, the interaction is dominated by π–π and π–cation stackings and two Watson–Crick-like base readouts (U2708 and G2711) with the protein main chain (Fig. 4c). Uridine U2708, which points into the H59 RNA loop in structures of vacant ribosomes (Supplementary Movie 2), is bulged out and accommodated in a binding pocket created by residues of Ebp1 helix α6 and its N-terminal loop (Fig. 4d; Supplementary Fig. 8b). While the uridine base is sandwiched between Asn254 and Phe266, its Watson–Crick edge is perfectly read out by the protein main chain (Tyr255 and Leu257). The interaction is completed by Ser267 hydrogen bonding to the ribose moiety. An almost identical interaction has been observed as a major determinant in the Arx1–pre-60S interaction^14^, although the base-specific readout was not described (Supplementary Fig. 8c). As this specific readout is mediated by the main chain, the conservation of the binding pocket is not obvious in respect to the MetAP-2 family; however, the overall features of this docking region are preserved (Supplementary Fig. 1). The two subsequent nucleotides of H59 (C2709 and C2710) are stacked on top of the loop and held in place by the capping π–cation stacking with Arg271 (Fig. 4c).

Upon H59 remodeling, nucleotide G2711 undergoes the largest movements, and like U2708, it is fixed in a base-specific binding pocket at the N-terminal end of Ebp1 helix α6 in the docked position (Fig. 4e). Unlike the binding site for U2708, G2711 is accommodated in a composite pocket formed by Ebp1 and helix α3 of eL19. Here, the base is stacked between Arg263 of Ebp1 and Gln40 of eL19, and the Watson–Crick readout is mediated by the Ebp1 main chain (Leu257 and Lys258). The guanine base is further hydrogen bonded to side chains within helix α3 of eL19 (Ser37 and Gln39), which itself also contacts Ebp1 directly (Asp34 and Asp36). The respective nucleotide is missing in yeast, although an Arx1-eL19-binding pocket would be present in principle. Finally, as a consequence of the H59 re-arrangement, the rRNA helix is rolled onto helix α3 of eL19, which by itself establishes a ribosome internal specific base recognition of guanine G2707 by Glu47 complemented by charged interactions (Lys43 and Lys46) (Fig. 4f).

Taken together, the interaction of the Ebp1 insert domain with H59 of 28S rRNA defines this eukaryotic rRNA expansion segment as a dynamic adaptor for PA2G4/MetAP-2-fold proteins at the ribosomal tunnel exit. The ribosomal interaction site (H59 and eL19) as well as the respective Ebp1/MetAP-2 regions are structurally conserved in mammals (rmsd of 1.7 Å for 287 Cα-atoms; Supplementary Fig. 1), indicating that binding of MetAP-2 and possibly other factors might employ the conformational plasticity of H59 in a similar manner.

## Discussion

**Fig. 5:**
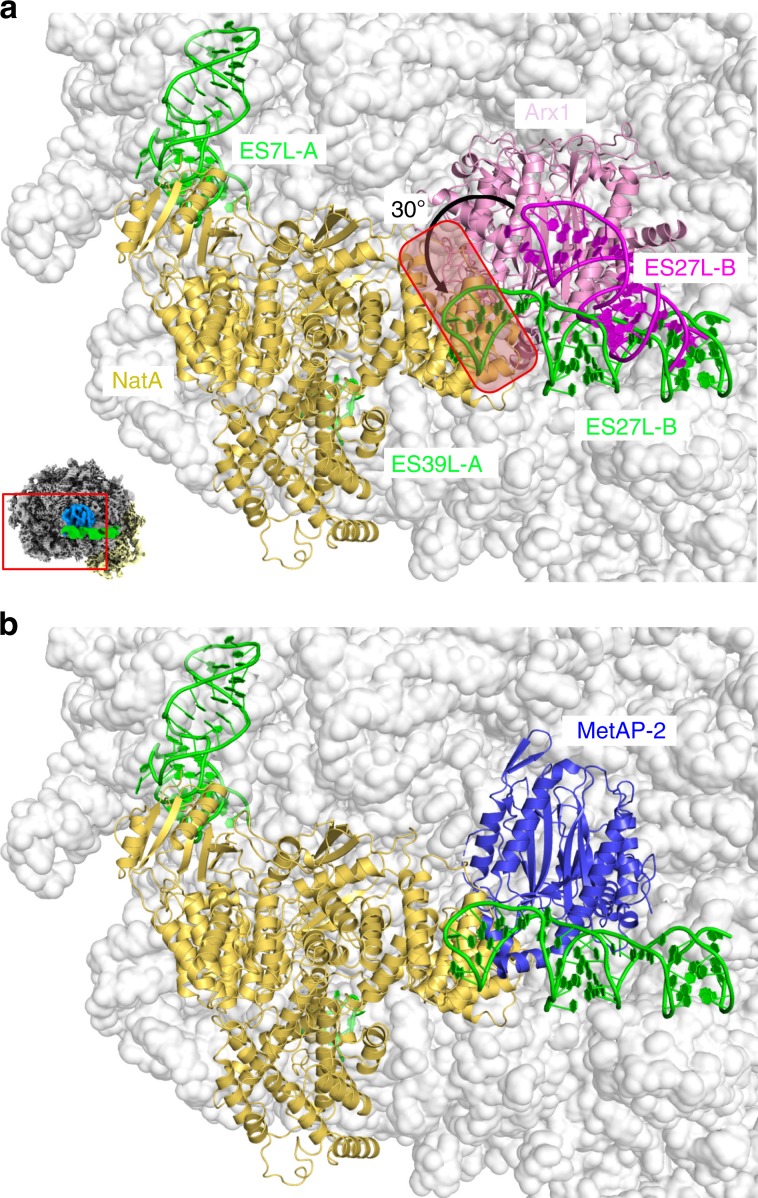
PA2G4 proteins as MetAP-2 structural homologs. **a** Structure of the NatA–ribosome complex from yeast^25^ (PDB ID: 6HD7) superposed with Arx1–ES27L (in pink tones) taken from the Arx1–ribosome complex^14^ (PDB ID: 5APN). ES27L-B was built de novo in this study. The C-terminal region of Arx1 clashes (boxed region) with the Naa10 subunit, and ES27L-B conformation is different (arrow). The view is identical to Fig. 1c top panel, and as indicated by the small representation in the corner. **b** Proposed model for simultaneous MetAP-2/NatA binding in eukaryotic co-translational protein modification based on the yeast NatA complex and the human MetAP-2 X-ray structure^54^ (PDB ID: 1BN5) superposed on Arx1.

The structure of the Ebp1–ribosome complex revealed the importance of plastic rRNA expansion segments in specific ligand recruitment (or vice versa) at the ribosomal tunnel exit. Besides helix H59, the tentacle-like ES27L was identified in its ES27L-exit conformation to serve as a major rRNA docking site for Ebp1. In a most recent cryo-EM reconstruction of the yeast ribosome–NatA complex, the trimeric NatA complex (Naa10/Naa15/Naa50) was also shown to recruit ES27L in the exit position by binding to the closing tetraloop^25^ adjacent to the *cs2* region. NatA was described with a unique mode of ribosome interaction, contacting ESs in three out of four binding patches. Interestingly, the NatA-binding site is directly adjacent (shifted toward ES7a and ES39a) to the Ebp1/Arx1-binding site, with only the PA2G4-specific C-terminal extension of the MetAP-fold clashing (Fig. 5a). Thus, while according to structural comparison, Ebp1 and NatA binding are likely mutually exclusive, concomitant MetAP/NatA binding would be possible^25^ (Fig. 5b). Moreover, the suggested minimal NC length of 50 amino acids in order to reach the Naa10 catalytic site^25^ is only ten residues longer as found for the MetAPs, strongly indicating a direct handover mechanism. The distal end of ES27L-B undergoes a conformational transition from the Arx1- (MetAP-) to the NatA-bound state with a 30° bending (translation of 30 Å of the tetraloop) (Fig. 5a). Therefore, the MetAP/ES27L-B contact is likely to be disrupted upon NatA binding, which potentially prepares subsequent dissociation of MetAP.

**Fig. 6:**
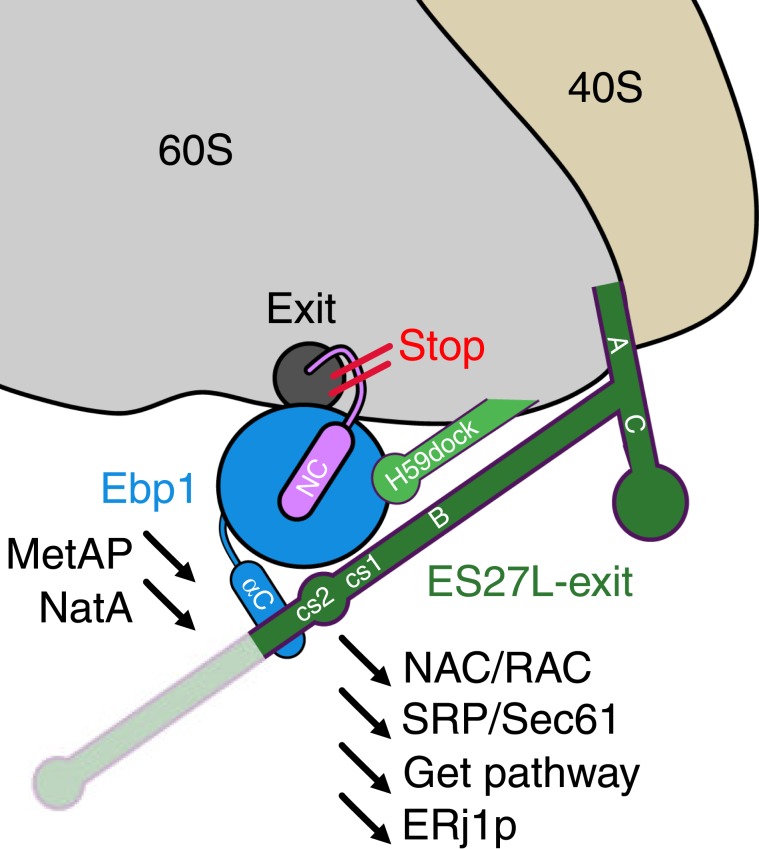
Model for Ebp1 function in health and disease. The recruitment of Ebp1 (p48 isoform) to the ribosomal tunnel exit excludes NC-associated factors like modifying enzymes (MetAPs, Nats), chaperones (RAC/NAC), and targeting factors (SRP/Sec61, Get pathway). Protein biogenesis is stopped or at least slowed down as the NC is sterically hindered from exiting the ribosomal tunnel.

The structural homology of the PA2G4 member Ebp1 with the MetAP family and the resulting competition for ribosome binding raises questions regarding physiological readout (schematized in Fig. 6) and potential pathological implications. Evidently, binding of Ebp1 and MetAPs or Nats is mutually exclusive, and persistent Ebp1 binding to the ribosomal tunnel exit would consequently prevent any co-translational protein modifications by either the MetAPs or the NATs with severe effects on the cellular proteome. Whether also protein synthesis per se is arrested upon Ebp1 binding is more challenging to address on a structural basis. The plurality of ribosomal protein and rRNA-binding events within the Ebp1–ribosome complex, in particular the arrest of conformational dynamics of ES27L, might induce conformational changes in the ribosome that permit long-scale signal transfer toward the peptidyl transferase center (PTC) and thus impact on translational kinetics. However, our cryo-EM reconstruction does not indicate such a conformational signal transfer upon Ebp1 binding, as the conformation of ribosomal proteins and rRNA in the PTC remains unaltered compared with the vacant mammalian 80S ribosome. As observed upon focused 3D classification, the same holds true for conformational dynamics of the ribosomal small subunit, which recapitulates the canonical ratcheting and rolling motions of the translational elongation cycle also after Ebp1 binding, as well as for translational elongation factor binding, which seems not to be affected (Supplementary Fig. 9). Therefore, our cryo-EM data do not per se support a consequent mechanistic translation inhibition upon Ebp1 binding. However, during elongation, any NC would be sterically hindered in exiting the ribosome by the blockade via (or potential binding to) Ebp1 and the conformational fixation of ES27L in the exit position, which collectively might affect translation in an indirect way. Whether such stalled Ebp1–ribosome–NC complexes exist and if the Ebp1–ribosome interaction may alter in the presence of a NC remains to be addressed.

Ebp1 inhibits phosphorylation of initiation factor eIF2α^4^, and phosphorylation is likely to be regulated by the phosphorylation status of Ebp1 itself. As estimated from computational particle sorting during cryo-EM data processing (Supplementary Fig. 2), ~25% of our ribosomes purified from HeLa cells were already decorated with Ebp1, a fact which in previous studies has been either overlooked or not been commented on. This occupancy could be raised up to ~75% upon addition of recombinant Ebp1. In our studies, we cannot resolve any putative phosphorylation at the Ebp1 C-terminus, and especially that of Ser360. However, Ser360 is in direct vicinity to the phosphoribose backbone of the ESL27L-B *cs2* region (Fig. 2a, e), and a detrimental effect of its phosphorylation for ES27L binding can be assumed. This indicates that Ebp1 binding to mature ribosomes requires non-phosphorylated Ser360 and further C-terminal residues, as the subsequent Ebp1 helix αC penetrates deeply into the major groove of ES27L-B and engages in electrostatic interactions with the negatively charged phosphate backbone of the rRNA.

HeLa cells are derived from cervical cancer, and Ebp1 is well known as an essential regulator of cell growth, with the direction of regulation and effects on cancer progression still being debated (reviewed in ref. ^1^). This raises the question of whether Ebp1 binding to ribosomes is enhanced in HeLa cells compared with healthy cells or cells from other tissues. To this end, we compared the overall Ebp1 occupancy on ribosomes in HeLa cells to HEK293 cells derived from embryonic kidney cells, which at least by qualitative immunoblot analysis did not reveal any significant differences (Supplementary Fig. 10). Further, overall data analysis of gene expression profiling in different cancer cell lines indicates on average not more than about twofold elevation of Ebp1 expression over paired normal tissues (http://gepia.cancer-pku.cn/). The effect of Ebp1 on cell proliferation in cancer depends on its isoform: while the p48 isoform has pro-oncogenic effects, the p42 isoform is a tumor suppressor. Isoform p42 can directly bind to the ErbB3 receptor^40^, but lacks the 54 N-terminal residues of p48 that are involved in recognition of the *cs1* element of ES27L-B and are an integral feature of the MetAP-like fold. The p42 isoform could therefore have different properties in terms of overall conformation, folding and in particular ribosome binding. While experimental validation is missing, the truncated N-terminus of p42 indicates that binding to the ribosome might be impaired. Thus, Ebp1 binding to ribosomes is likely isoform-specific, and consequently might correlate with tumor progression. Dysregulation of translation is a frequent feature of fast proliferating cells, e.g., in neoplasia^41^, and thus an inhibitory effect of p48 on translation could have protective effects under such conditions. The correlation of the multiple (patho-)physiological and cellular functions with respective molecular mechanisms will be the next challenge in Ebp1-related research.

## Methods

### Sample preparation

The total human mRNA was extracted from HEK293-S3 cells using the Direct-zol RNA Miniprep kit (Zymo Research) and reversed transcribed into cDNA utilizing the Maxima first strand cDNA synthesis kit with dsDNase (Thermo Scientific). The coding sequence of the *EBP1* gene was amplified by PCR using the HEK cDNA as a template and the primers Ebp1NcoI_Fwd (CATGCCATGGCTTCGGGCGAGGACGAGCAAC) and Ebp1BamHI-Rev (CGGGATCCTCAGTCCCCAGCTTCATTTTCTTC). The ~1200 bp PCR product was digested with NcoI/BamHI restriction nucleases (NEB) and ligated into the pET24d-His_6_-linker-TEV (tobacco edge virus) resulting in the pET24d-His_6_-linker-TEV-Ebp1 plasmid.

Ebp1 was expressed from the pET24d-His_6_-linker-TEV-Ebp1 plasmid in *E. coli* Rosetta 2 cells (Novagen) using autoinduction medium^42^. Cells were cultured at 37 °C until reaching OD_600_ 0.6–0.8, upon which the temperature was shifted to 21 °C and expression was continued for 16 h. Cells were harvested and resuspended in lysis buffer (40 mM HEPES KOH pH 7.5, 1 M NaCl, 10 mM MgCl_2_, 10 mM KCl, 40 mM Imidazole, 0.02% 1-thioglycerol) supplemented with protease inhibitor cocktail (Roche) at 1× final concentration. Resuspended cells were lysed utilizing a microfluidizer (Microfluidics Corp.), and the lysate was cleared via centrifugation for 20 min at 20.000 × *g* and 4 °C. The cleared lysate was applied to Ni-IMAC (immobilized metal affinity chromatography) (GE Healthcare) and thoroughly washed with lysis buffer. Ebp1 was eluted in 20 CV using elution buffer (40 mM HEPES KOH pH 7.5, 1 M NaCl, 10 mM MgCl_2_, 10 mM KCl, 500 mM Imidazole, 0.02% 1-thioglycerol). The eluate was transferred to a 8.000 MWCO dialysis tubing (Spectrum Labs) including TEV protease at a final concentration of 40 µg/mL and dialyzed against dialysis buffer (20 mM HEPES KOH pH 7.5, 500 mM NaCl, 5 mM MgCl_2_, 5 mM KCl, 5 mM 2-mercaptoethanol) at 4 °C. TEV digested Ebp1 was isolated via reverse Ni-IMAC and in a final step purified via Superdex 75 (GE Healthcare) size-exclusion chromatography (SEC) equilibrated in SEC Buffer (20 mM HEPES KOH pH 7.5, 5 mM Mg(OAc)_2_, 175 mM KOAc, 1 mM tris(2-carboxyethyl)phosphin). Ebp1 containing fractions were pooled, snap frozen in liquid nitrogen, and stored at −80 °C until further use.

Human non-translating 80S ribosomes were isolated from HeLa cells in a protocol as described previously^43^ that we adapted from a large-scale setup^44^. Briefly, HeLa cells were grown in suspension cultures and harvested cells (1 × 10^8^ cells per 100 mL) were lysed with detergent. After clearing of the lysate from debris and membranes, ribosomes were purified via centrifugation through a sucrose cushion. The pellet was resuspended, treated with puromycin and the monosomes further purified in a sucrose gradient. The monosome peak was collected and concentrated to 1 mg/mL in the same physiological buffer as Ebp1 (SEC buffer), aliquoted and snap frozen in liquid nitrogen and stored at −80 °C. The typical yield is 1 mg per 1 × 10^8^ cells.

### Immunoblot analysis

HEK293 and HeLa cell lysates were separated on a 12.5% SDS-PAGE gel and transferred onto a Protran nitrocellulose membrane (Amersham). The membrane was stained with Ponceau-red S to detect the total protein and probed with antibodies against Ebp1 (N-terminus, ABE43, 1:1000, Merck Millipore). Ebp1 was detected with peroxidase-conjugated goat anti-rabbit IgG antibody (111-035-144, 1:5000, Jackson ImmunoResearch) and visualized with the ECL Western Lightning Ultra (Perkin Elmer), according to the manufacturer’s protocol.

### Grid preparation

Two batches of EM grids were prepared. The first batch used for collection of dataset 1 contained the in vivo pulled-out Ebp1–ribosome complex without addition of recombinant Ebp1. The second batch used for collection of dataset 2 contained the same purified ribosomes, but supplemented with recombinant Ebp1 (p48 isoform) to increase Ebp1 occupancy on the ribosomes. Right before freezing, Quantifoil Multi A holey carbon supported grids (Quantifoil, Multi A, 400 mesh) were glow-discharged for 10 s in oxygen atmosphere using a Solarus plasma cleaner (Gatan, Inc.). In total, 3 μL of freshly prepared samples (100 nM ribosomes without/with a eightfold excess of recombinant Ebp1) were directly applied to glow-discharged grids. Under a blot force of 0 at 100% humidity, the grids were blotted for 3 s with Whatman #1 filter papers using a Vitrobot Mark IV (FEI Company) operated at room temperature, and then immediately plunge-frozen in liquid ethane cooled with liquid nitrogen.

### Data collection

Cryo-EM data were acquired on a Titan Krios transmission electron microscope (Thermo Fisher/FEI Company) operated at an acceleration voltage of 300 keV. Two data sets were collected. The first dataset (dataset 1) was acquired on a K2 Summit direct electron detector (Gatan, Inc.) at an object pixel size of 1.07 Å in Latitude (Gatan Company) with a target defocus range of −1.5 to −3.0 µm. Micrographs were acquired using dose fractionation to record 40 frames per exposure with a dose rate of 0.9 electrons per Å^2^ per frame, resulting in a total dose of 39 electrons per Å^2^ per micrograph. The second dataset (dataset 2) was acquired on a K3 direct electron detector (Gatan, Inc.) at an object pixel size of 1.07 Å. Data were collected using the SerialEM software package with a target defocus range of −0.5 to −2.0 µm. Micrographs were acquired using dose fractionation to record 20 frames per exposure with a dose rate of 1.85 electrons per Å^2^ per frame, resulting in a total dose of 37 electrons per Å^2^ per micrograph.

### Image processing

All steps of image processing are summarized in a visual flow chart (Supplementary Fig. 2). Both data sets were initially processed separately, but following the same workflow. All processing steps were performed with the RELION 3.0-beta software package, unless stated otherwise^45^. Movie stacks were motion-corrected using MotionCor2 with the number of patches set to 5 × 5^46^. Estimation of contrast transfer function (CTF) parameters was performed with Gctf on the motion-corrected micrographs^47^. To generate reference templates for auto-picking, ~500 ribosomal particles were manually selected from the micrographs and subjected to unsupervised two-dimensional (2D) classification into ten classes. Classes depicting 80S ribosomes were used for auto-picking, resulting in 162,886 particles from dataset 1, and 81,482 particles from dataset 2. The particles were extracted at a pixel size of 4.28 Å in boxes of 128 × 128 pixels and subjected to 3D classification using a human 80S ribosome filtered to 40 Å resolution as an initial reference. Only ribosomal classes depicting high-resolution structural features were retained for further processing, yielding 70,414 and 73,759 particles from dataset 1 and dataset 2, respectively. A second round of 3D classification was focused on the polypeptide exit tunnel to enrich ribosomal particles with clear density for Ebp1 and ES27L, yielding 21,743 and 55,177 particles from the first and second dataset, respectively. For this purpose, we generated a binary mask encompassing only Ebp1 and ES27L from one of the 3D classes via manual segmentation of the density in UCSF Chimera^48^ and used it as a reference mask during 3D classification. Sampling was switched off during this second round of 3D classification, and optimal translations and rotation from the first round of 3D classification were used for each particle. The retained particles were re-centered, extracted at a pixel size of 1.52 Å in boxes of 360 × 360 pixels and subjected to 3D auto-refinement. After 3D auto-refinement, particles were subjected to CTF refinement using standard parameters (plus dataset-wise beam tilt estimation) and Bayesian particle polishing^49^ using a training set of 5000 particles.

At this point, we joined polished particles from both data sets and subjected them to 3D auto-refinement. In the next step, a third round of 3D classification was focused on Ebp1–ES27L, again keeping particle positions and orientations fixed according to optimal parameters determined during the preceding 3D auto-refinement run. The remaining 34,467 particles with optimal density for Ebp1–ES27L were selected and subjected to a final round of 3D auto-refinement, which served as basis for 3D multibody refinement. In a first run, we split the 80S–Ebp1 density into two separate segments (“2-body”), comprising the 40S ribosomal subunit and the 60S ribosomal subunit plus Ebp1, respectively, to compensate for intersubunit rotation of the ribosomal subunits. This approach resulted in density segments at 3.55 Å (40S) and 3.3 Å global resolution (60S-Ebp1) after post-processing using the subunit masks also used for multibody refinement. Lower local resolution and smeared out density for peripheral regions of Ebp1 and ES27L density segments suggested conformational mobility of Ebp1 and ES27L independent from the 60S subunit. We thus subjected particles to a second round of 3D multibody refinement, in which we split the 80S–Ebp1 complex into three independently moving segments (“3-body”): the 40S subunit, the 60S subunit and the Ebp1–ES27L segment. This resulted in density segments at 3.55 Å (40S), 3.3 Å (60S), and 5.7 Å global resolution (Ebp1–ES27L) after post-processing using the masks also used for multibody refinement. Interpretable features in particular for ES27L greatly improved using this approach.

In an attempt to improve resolution of the 60S ribosomal subunit for H59 model building, we repeated the initial processing steps for the pre-selected particles extracted at full spatial resolution (1.07 Å pixel size, box size of 512 × 512 pixels). After 2-body multibody refinement, global resolution of the 60S-Ebp1 segment after post processing was comparable (3.3 Å), but interpretability of the density improved because of the finer sampling.

For analysis of ES27L conformational mobility, we prepared a binary mask including exclusively the ES27L density projecting from the ribosomal core towards Ebp1 and used it for a focused 3D classification of the joined set of 76,920 particles. Again, particle positions and orientations were kept fixed according to optimal parameters determined during the preceding 3D auto-refinement run. Three out of the ten resulting classes represented well-defined states of ES27L in slightly different conformations. Particles belonging to these classes were selected and independently subjected to one round of 3D auto-refinement and 2-body multibody refinement. After post-processing, global resolution of the 60S-Ebp1 density segment was estimated to be approximately 4.5 Å for all three classes, but resolution for ES27L was clearly lower and the density maps were filtered according to local resolution for interpretation.

All resolution estimates were performed according to the “gold standard” FSC criterion of independently refined half maps (FSC = 0.143) within RELION. Local resolution was estimated using RELION’s local post-processing implementation. The B-factor values used during local resolution filtering were guided by B-factors fitted during post processing of the various density segments, and ranged from −50 to −200 Å^2^.

### Atomic model building and refinement

As starting point, the high-resolution cryo-EM structure of the human 80S ribosome solved at 2.9 Å resolution^22^ (PDB ID: 6EK0) was rigid body fitted into the cryo-EM density map, computed at full spatial resolution in UCSF Chimera^48^. All further atomic model building was then performed in COOT^50^. The 1.6 Å Ebp1 X-ray structure^5^ (PDB ID: 2Q8K) was manually transferred into the corresponding cryo-EM density at the ribosomal tunnel exit and real space refined by rigid-body placement. Restrained real-space refinement and validation was performed with the PHENIX suite^51,52^. In a first step, we optimized the fit for Ebp1 and its conformationally unchanged contact partners within the inner ring of the tunnel exit (eL19, uL23, uL24, and uL29 as well as the 5.8S rRNA (helix 24) and the 28S rRNA (helices H24, H47, H53)). In a second step, helix H59 of 28S rRNA was remodeled and refined together with the already refined model. For model building of the contact site between Ebp1 and ES27L-B, we used the cryo-EM density obtained after “3-body” refinement. An ideal A-RNA double helix was placed in the ES27L-B region interacting with Ebp1, and GA and UG mismatches were manually optimized for favorable hydrogen bonding at their Watson–Crick edges. The sequence register was defined by a combination of structural information for ES27L-B from yeast^25^, apparent bulging of A3252 and the defined distance to ES27L-B emanation from the previously built and well-defined ES27L-A^22^. The C-terminal helical extension of Ebp1 was subsequently built in the rod-shaped density within the widened major groove of ES27L-B, and the sequence register was deduced from the adjacent C-terminus of the Ebp1 X-ray structure. Finally, the remainder of ES27L-B towards ES27L-A was built accordingly to the ES27L–Ebp1 interaction region into one of the cryo-EM reconstructions obtained after 3D classification focused on ES27L, and the whole model was subjected to a final round of refinement and validation in PHENIX. Figures were prepared with programs UCSF Chimera^48^ and PyMOL^53^.

### Reporting summary

Further information on research design is available in the Nature Research Reporting Summary linked to this article.

## Supplementary information

Supplementary Information

Description of Additional Supplementary Information

Supplementary Movie 1

Supplementary Movie 2

Reporting Summary

## Data Availability

The following cryo-EM densities for the Ebp1–ribosome complex have been deposited in the EMDataBank: Ebp1-60S segment at full spatial resolution from 2-body multibody refinement (EMD-10344); Ebp1–ES27L segment from 3-body multibody refinement (EMD-10609); Ebp1-60S segment from 2-body multibody refinement after sorting for ES27L conformation (EMD-10608). The atomic coordinates for Ebp1 and interacting ribosomal components have been deposited in the RCSB with accession ID 6SXO. For visualization of the model in context of the entire human 80S ribosome, we recommend to superpose our atomic coordinates to the structure of the human ribosome solved at 2.9 Å resolution (PDB ID 6EK0)^22^, which is virtually identical except for the Ebp1-interacting region. Other data are available from the corresponding authors upon reasonable request.
