# Supplementary material for: MetAP-like Ebp1 occupies the human ribosomal tunnel exit and recruits flexible rRNA expansion segments
Source: Nat Commun. 2020 Feb 7;11:776. doi: 10.1038/s41467-020-14603-7 (PMC7005732; doi:10.1038/s41467-020-14603-7)
Supplement: Supplementary file 1 — Supplementary Information [file 41467_2020_14603_MOESM1_ESM.pdf]

Supplementary information

MetAP-like Ebp1 occupies the human ribosomal tunnel exit and recruits flexible rRNA expansion segments

Klemens Wild et al.

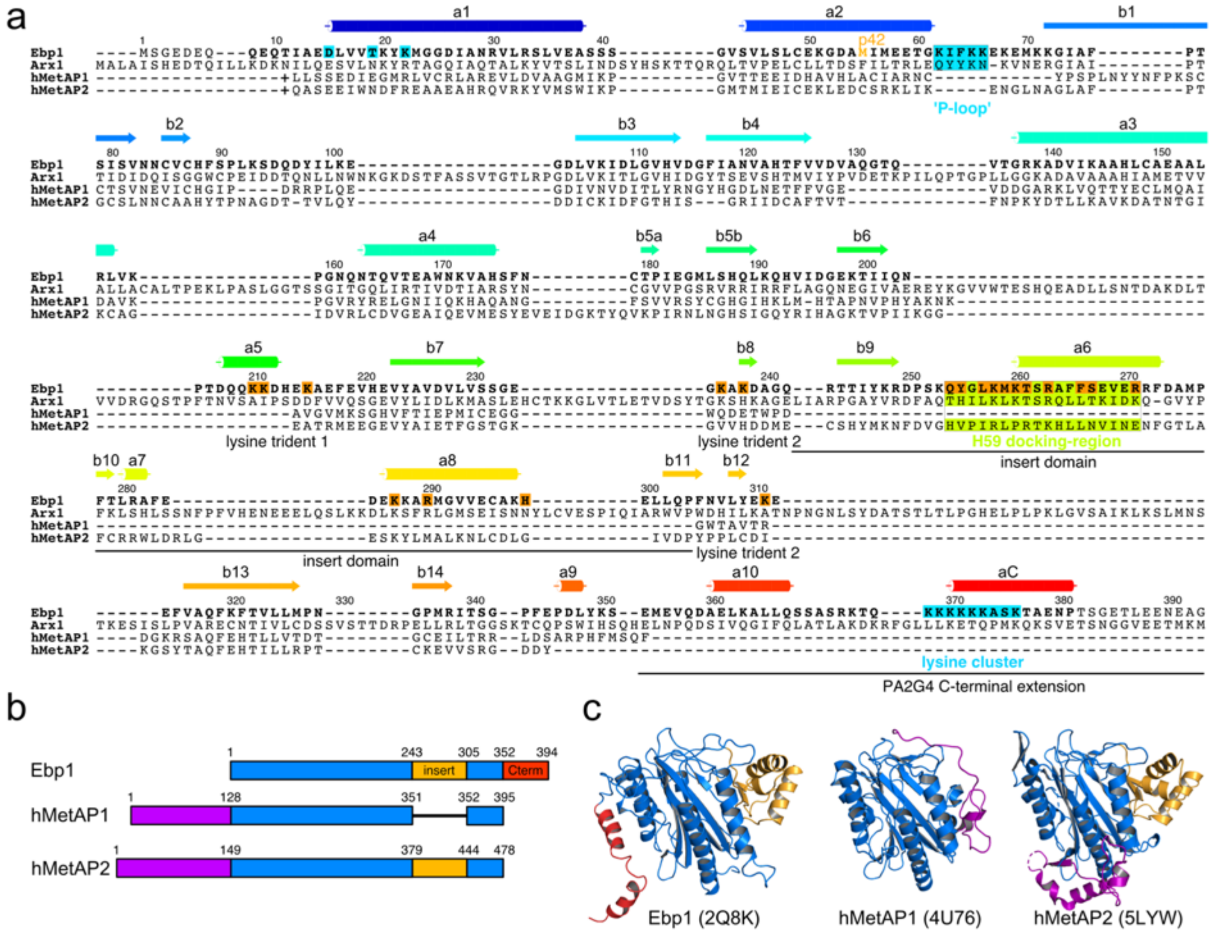

Supplementary Figure 1

Sequence and structure comparison of PA2G4 and MetAP family members.

a, Structure-based sequence alignment of human Ebp1 with its yeast homolog Arx1 (PDB ID: 5APN) of the PA2G4 family and with human MetAP1 (PDB ID: 2B3K) and MetAP2 (PDB ID: 5LYW). Numbering and secondary structures are given for Ebp1 as determined at high resolution[1] (PDB ID: 2Q8K) and for helix αC according to this study (built residues are in bold type). Ebp1 residues interacting with the ribosomal tunnel exit are highlighted in red and with rRNA ES27L-B in blue. The PA2G4-specific ‘P-loop’ contacting the *cs1* element of ES27L and the docking region (green) binding to H59 in the docked position are boxed. **b**, Scheme for domain organization of Ebp1, hMetAP1 and hMetAP2 with sequence numbering. **c**, Respective X-ray structures to (b) in the same color code. PDB IDs are given. All models are shown in the same view.

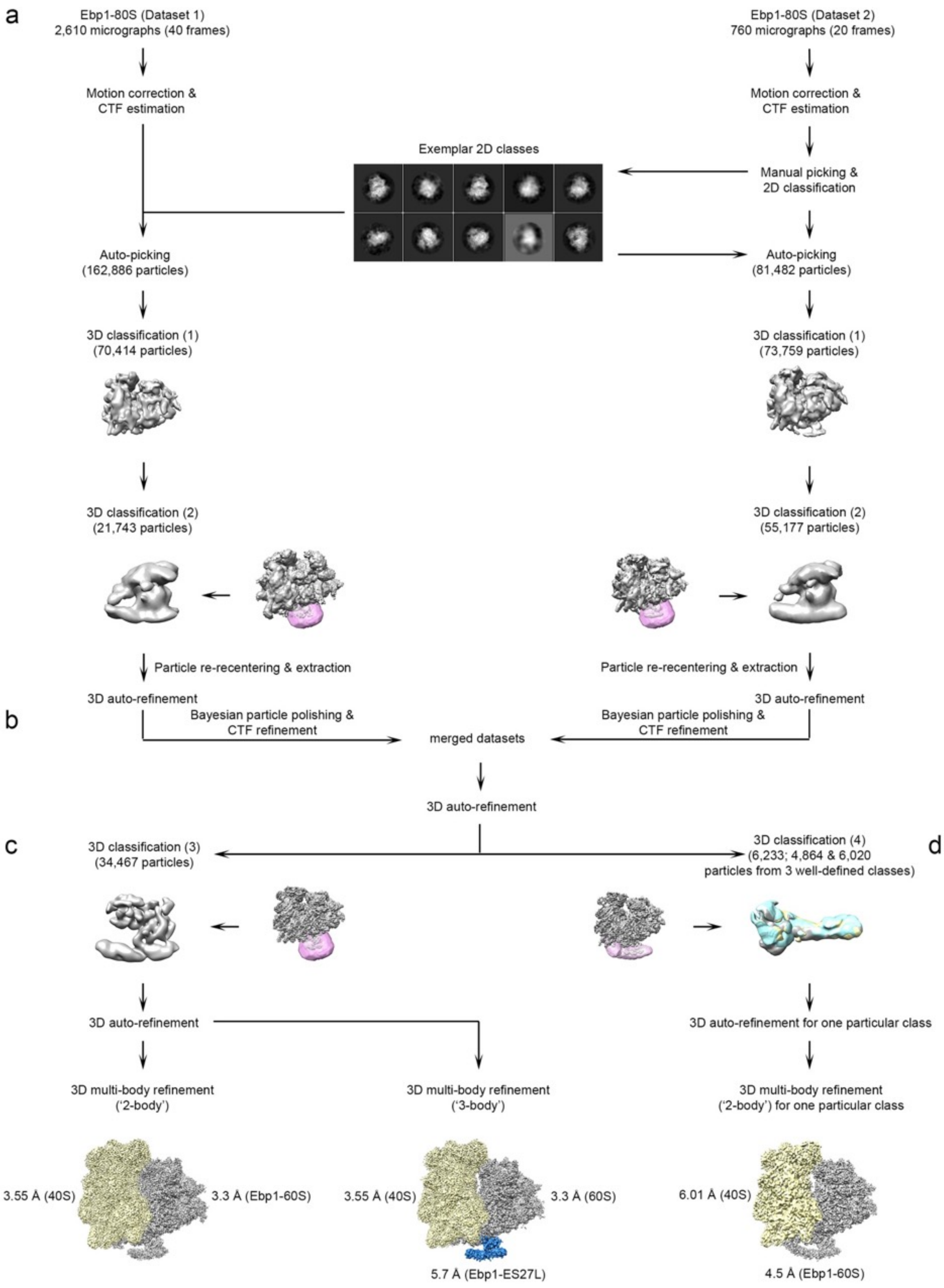

Supplementary Figure 2

Cryo-EM data processing workflow.

a, Two datasets were acquired and initially processed separately. Dataset 1 was acquired on the *in vivo* pulled-out Ebp1-ribosome complex without addition of recombinantly purified Ebp1. For dataset 2, the sample was supplemented with recombinantly purified Ebp1 to increase Ebp1 occupancy on the ribosome. Auto-picked particles were subjected to two consecutive rounds of 3D classification to reject false positive particles (1) and particles with no density for Ebp1 and ES27L (2). **b**, Retained particles were subjected to CTF refinement and Bayesian particle polishing, subsequently merged and subjected to 3D auto-refinement. **c**, Particles were subjected to another round of 3D classification focused on Ebp1 and ES27L (3) and the retained particles were subjected to either 2-body or 3-body multibody refinement. Segments used in the multibody refinement runs are color-coded. Resolution obtained for the separate segments after post-processing is given. **d**, Particles after merging of the datasets were subjected to another round of 3D classification focused on ES27L, yielding three classes with defined density for ES27L. One of the classes was subjected to 3D auto-refinement and 2-body multibody refinement.

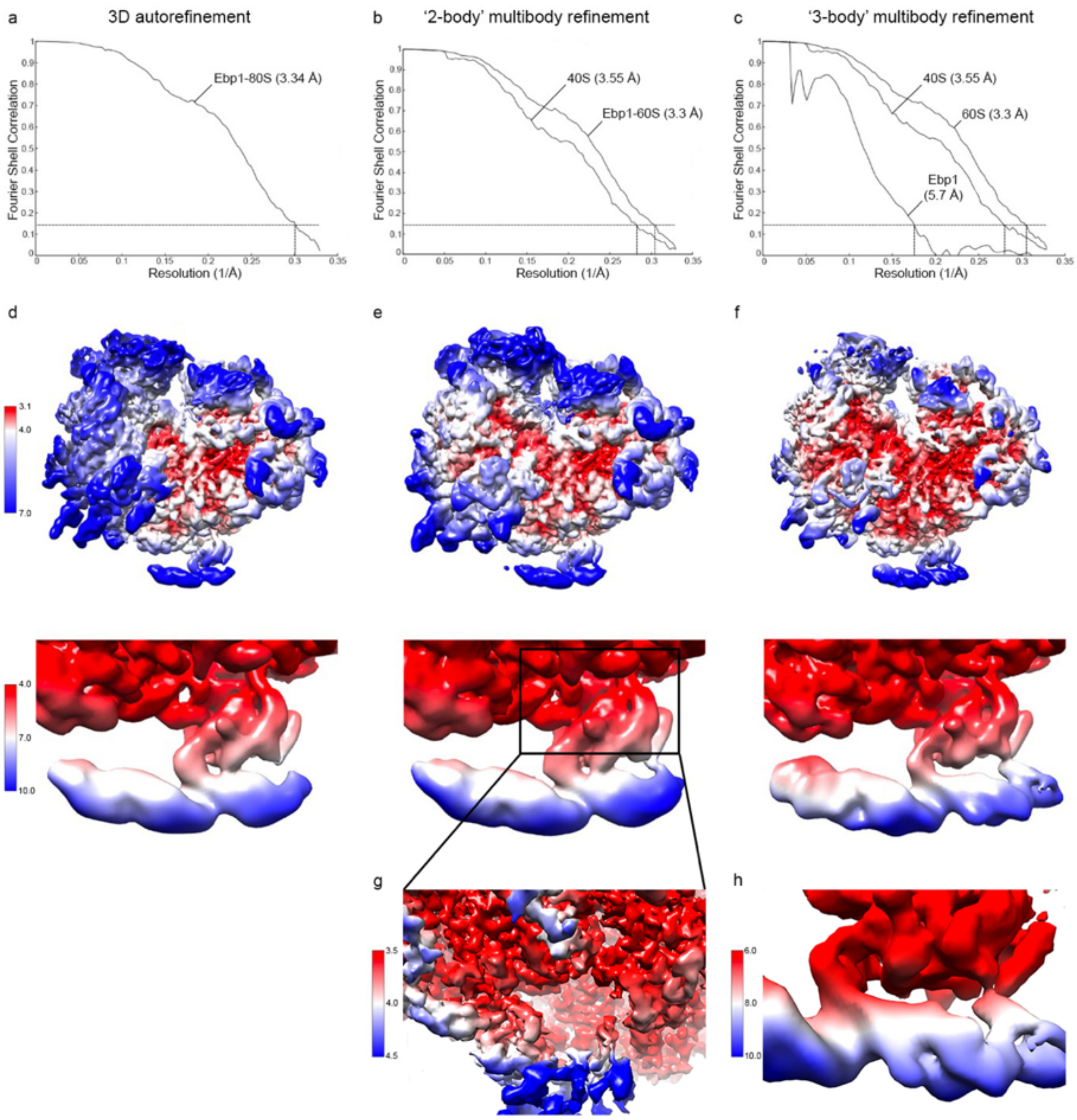

Supplementary Figure 3

Resolution estimates for the Ebp1-ribosome complex.

a-c, Fourier shell correlation (FSC) curves between two independently refined half sets of the data ('gold standard'). FSC curves are shown for the 3D auto-refinement (a) and the 2-body' (b) or '3-body' multibody refinement approaches (c). Determined resolution according to the FSC = 0.143 criterion is given for each curve. **d-f**, Lowpass-filtered composite cryo-EM reconstructions after 3D auto-refinement (d) and '2-body' (e) or '3-body' multibody refinement (f), colored according to local resolution as indicated. Lower row: zoom on the Ebp1-ES27L segment of the complex. Color-coded local

resolution range in the upper and lower row are not identical. **g-h,** Close-up views on the interface between Ebp1 and the ribosomal tunnel exit (g; from ‘2-body’ multibody refinement) or Ebp1 and ES27L (h; from ‘3-body’ multibody refinement). Densities are filtered and colored according to local resolution. The scale for coloring was chosen to cover only the relevant local range of resolution.

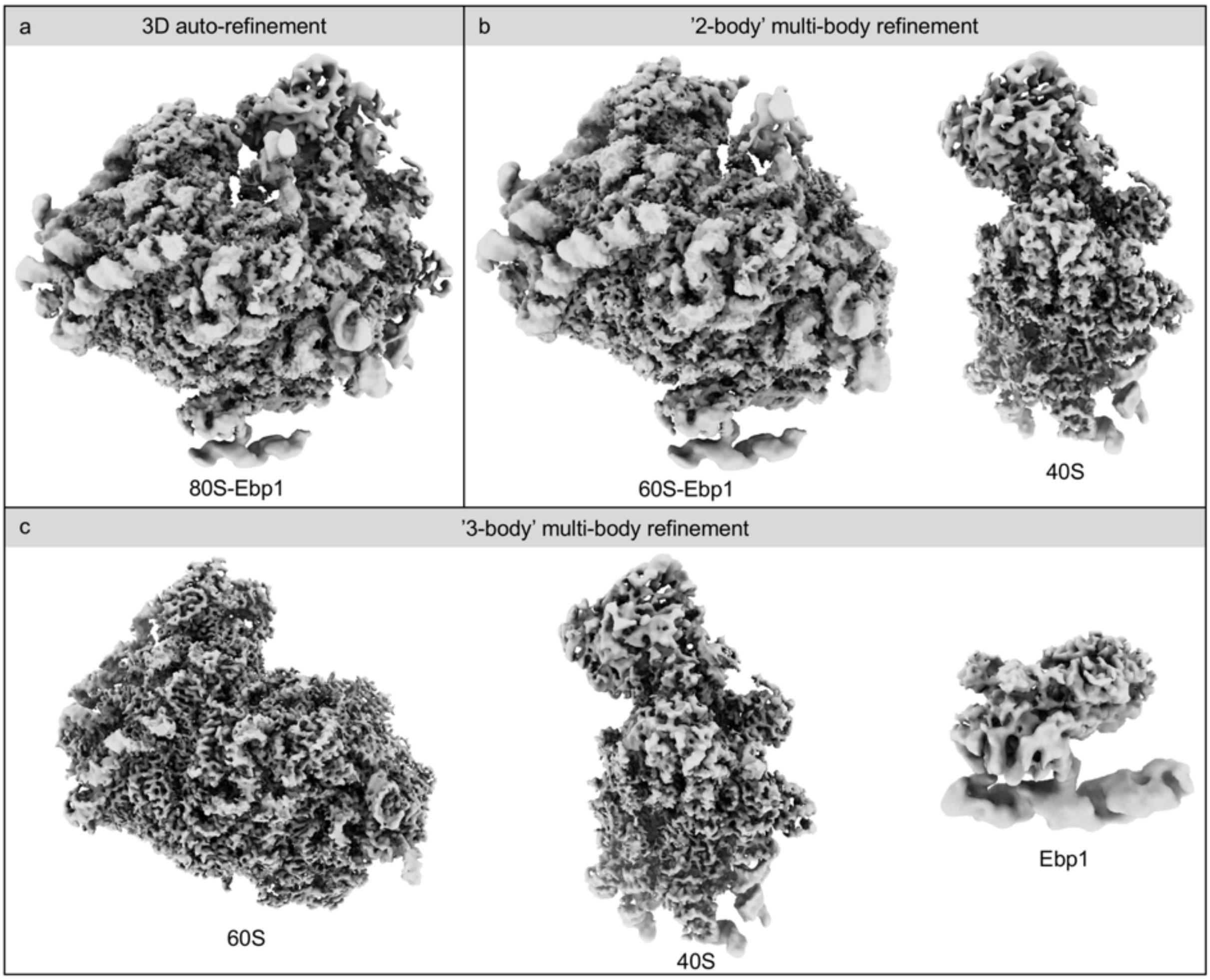

Supplementary Figure 4

Gallery of cryo-EM density segments obtained from the three individual refinement approaches.

Local resolution-filtered cryo-EM density segments obtained after 3D auto-refinement (a), ‘2-body’ multibody refinement (b) and ‘3-body’ multibody refinement (c). The interface between Ebp1 and the ribosomal tunnel exit was analyzed using the 60S-Ebp1 segment after ‘2-body’ multibody refinement (b), while the interface between Ebp1 and ES27L was analyzed using the Ebp1 segment after ‘3-body’ multibody refinement (c). Please note that Ebp1 in panel (c) is not shown to scale.

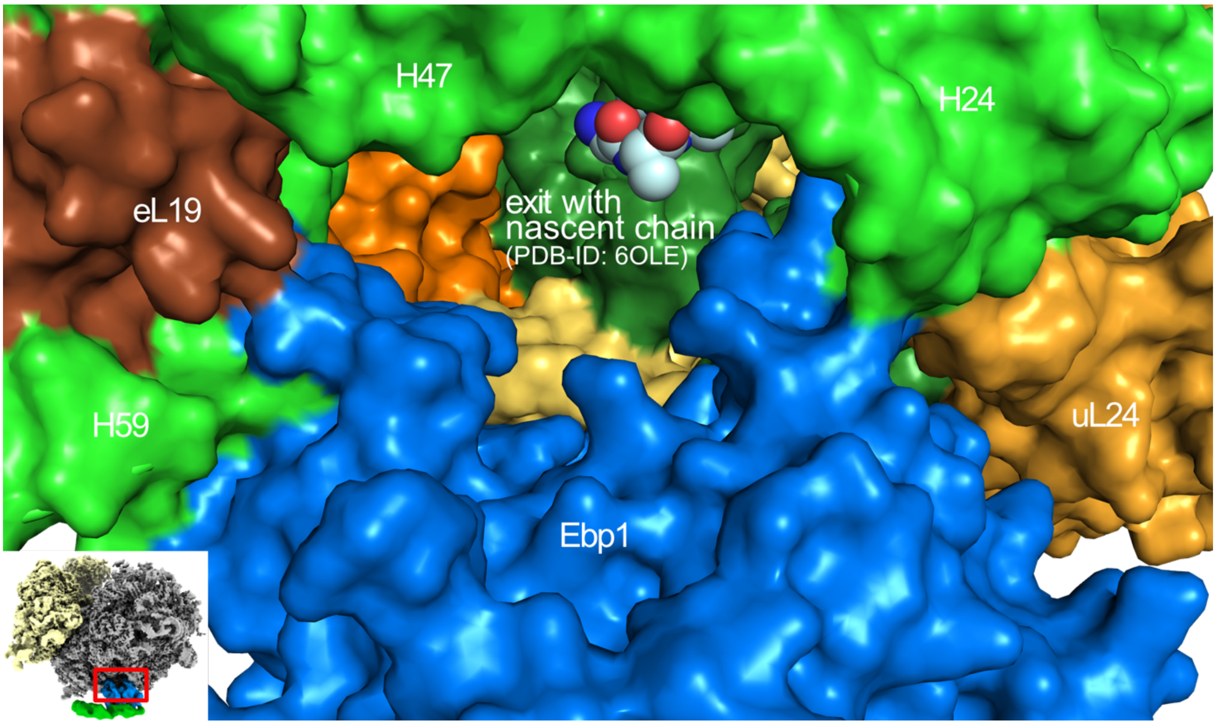

Supplementary Figure 5

Lateral gap between Ebp1 and the ribosomal tunnel exit.

While Ebp1 blocks the entire tunnel exit for any globular nascent chain binding factor, a small lateral gap is present that still would allow a nascent chain to pass by (nascent chain model from PDB-ID 6OLE[2]). The gap is lined by Ebp1, eL19, and 28S rRNA helices H24 and H47. Proteins and RNA fragments are shown in a surface view in the same colour code as in Fig. 1c. The view corresponds to Fig. 1c (bottom) rotated vertically by 180°. Overall orientation as indicated by the small representation in the corner.

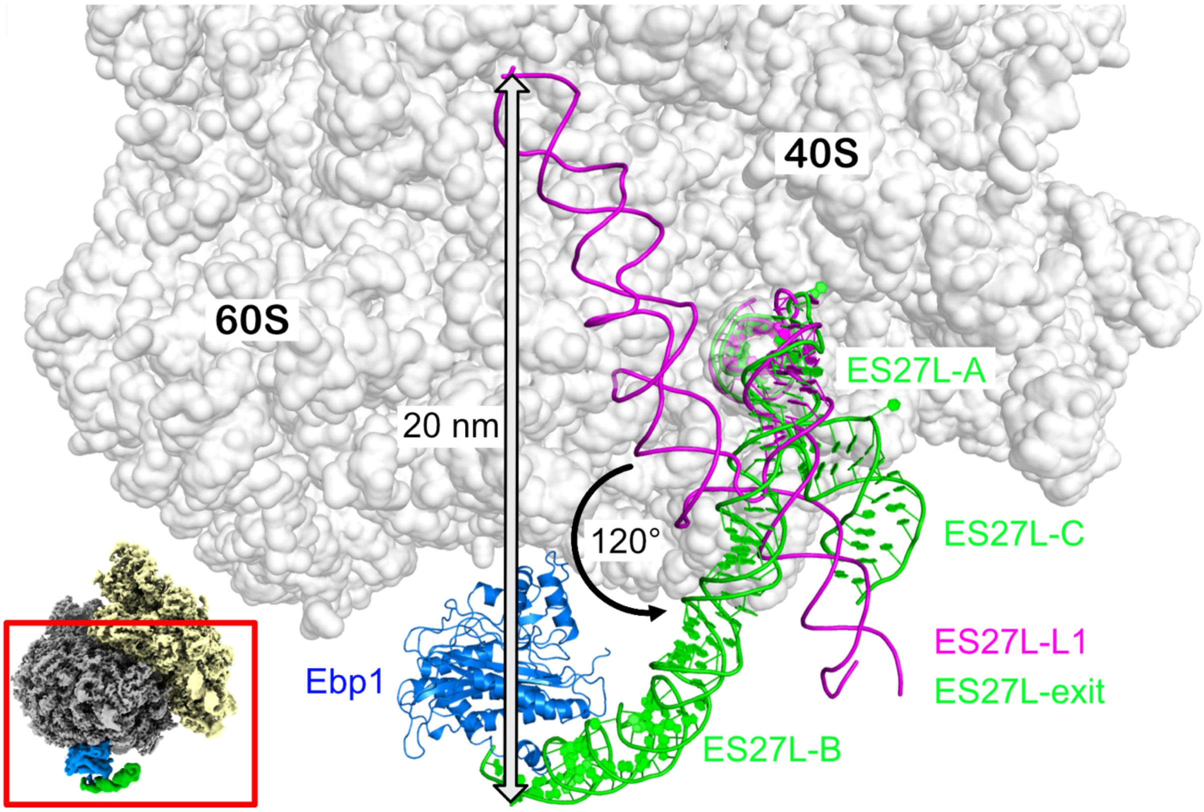

Supplementary Figure 6

L1- and exit-position of human expansion segment ES27L.

Cartoon representations for the atomic model of the ES27L transition from the ES27L-L1 position as found in vacant human ribosomes[3] (PDB-ID: 4V6X, only backbone built for ES27L-BC) to the ES27L-exit position in the Ebp1-ribosome complex. Overall orientation as indicated by the small representation in the corner.

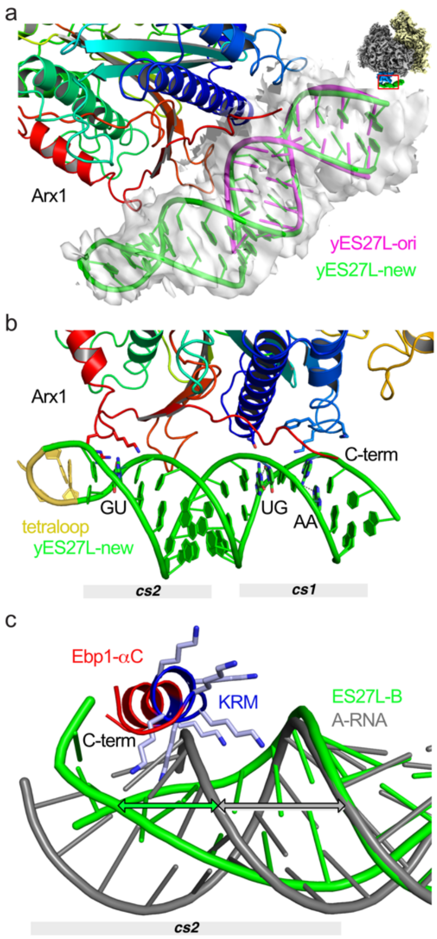

Supplementary Figure 7

The Arx1-ES27L interaction in yeast and A-RNA widening.

a, The yeast Arx1-ES27L interaction (rainbow/magenta) as part of the Arx1-ribosome complex[4] (PDB ID: 5APN). The distal end including the closing tetraloop of ES27L-B (green) is newly built into the cryo-EM density (EMD-3152). Overall orientation as indicated by the small representation in the corner. **b**, Details of the Arx1-ES27L contact. Contacts at ES27L *cs1* are almost identical to the Ebp1 ribosome contact. The *cs2* contact is different and the C-terminus of Arx1 does not enter the major groove, which is not widened as in the Ebp1-ES27L contact. **c**, The widened major groove including the AG/GA tandem purine mismatch of human ES27L-B *cs2* within the Ebp1-ribosome complex. The C-terminal helix (αC) of Ebp1 penetrates into the groove with its lysine-rich motif (KRM). Widening in respect to A-RNA is indicated with arrows.

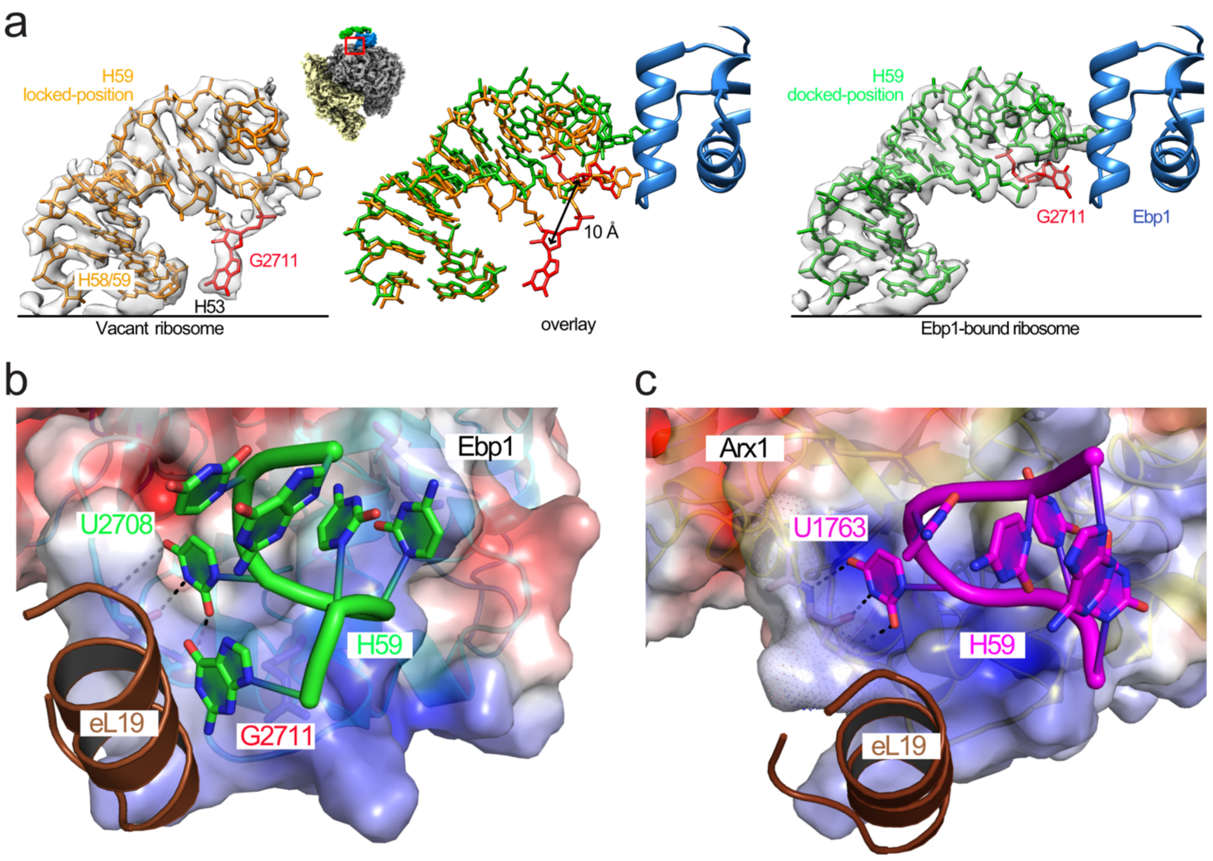

Supplementary Figure 8

The dynamic rRNA helix H59 adaptor.

a, Cryo-EM densities and atomic coordinates for human 28S rRNA helix H59 in the locked position on the vacant ribosome[5] (PDB ID: 6EK0) (left) and in the docked position within the Ebp1-ribosome complex (right). The overlay (central panel) highlights the movement of G2711 (depicted in red in all panels). Overall orientation as indicated by the small representation. **b**, Electrostatic surface potential (±5 kT, blue: positive, red: negative) for Ebp1 around the H59 docking-region including helix α6. The Watson-Crick readout of U2708 by the Ebp1 protein main chain is highlighted (dashed lines). **c**, The Arx1-H59 interaction within the Arx1-ribosome complex[4] (PDB ID: 5APN). The Watson-Crick readout of the uridine (U1763) in the closing loop is identical as in the human Ebp1-ribosome complex. A bulged-out guanine respective to G2711 in metazoan H59 is not present in yeast.

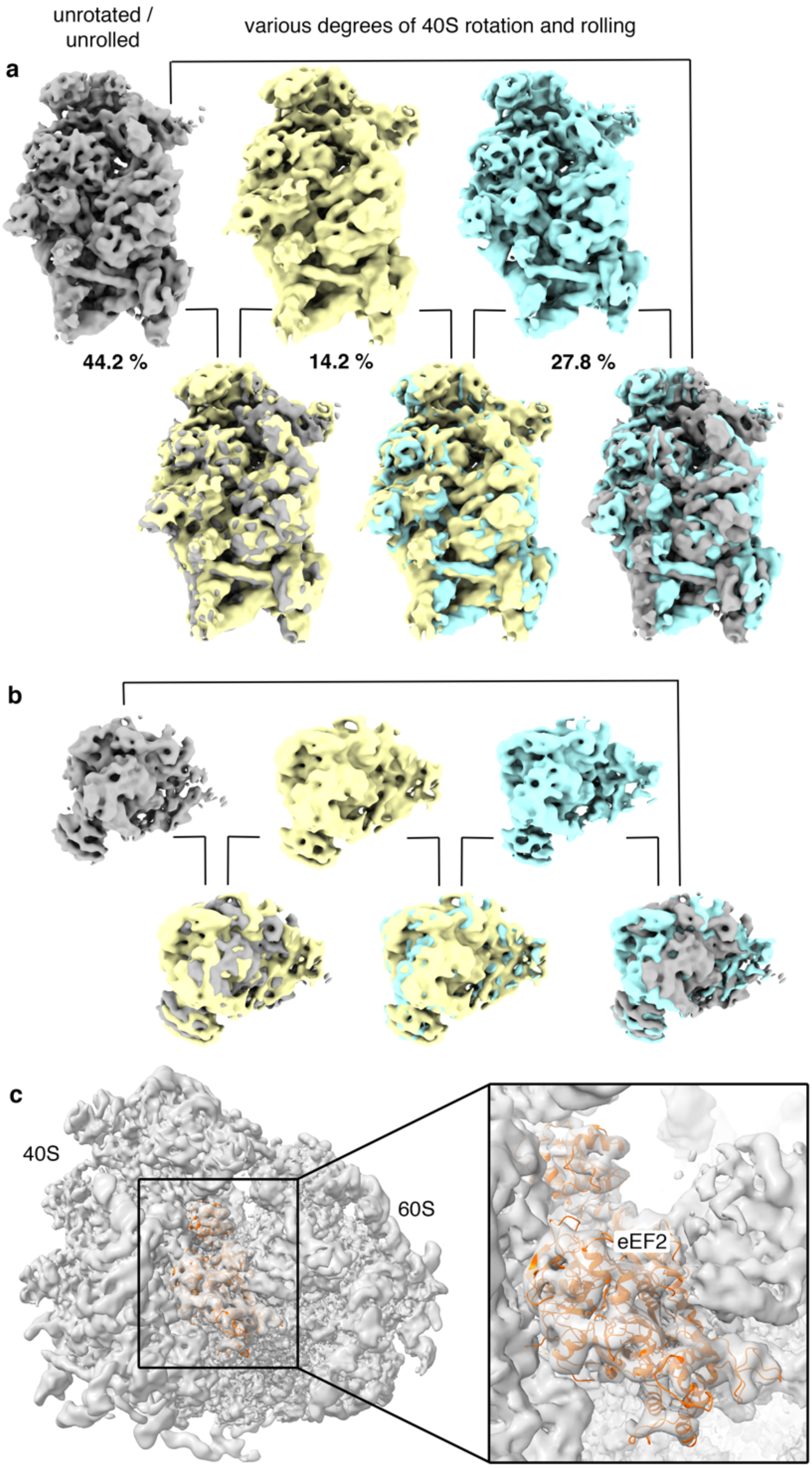

Supplementary Figure 9

Ebp1-binding does not restrict conformational dynamics of the ribosome or translational elongation factor binding.

a, Frontal view on the 40S ribosomal subunit. Upper row: most abundant 3D classes after classification focused on the 40S ribosomal subunit. Class abundance is given. Lower row: 3D classes were superposed as indicated for comparison of the conformational states. **b**, Same as in (a) but rotated by 90°. Only the 40S subunit head is shown. **c**, 3D classification focused on the ribosomal factor binding site identifies a subpopulation of Ebp1-containing particles (23.1%) with defined additional density at the ribosomal stalk base. The local resolution filtered cryo-EM density after 3D auto-refinement is shown (transparent grey). Rigid body docking of an atomic model for eukaryotic elongation factor 2 (eEF2; PDB: 6MTD; orange) identifies this particle population as eEF2-ribosome complexes.

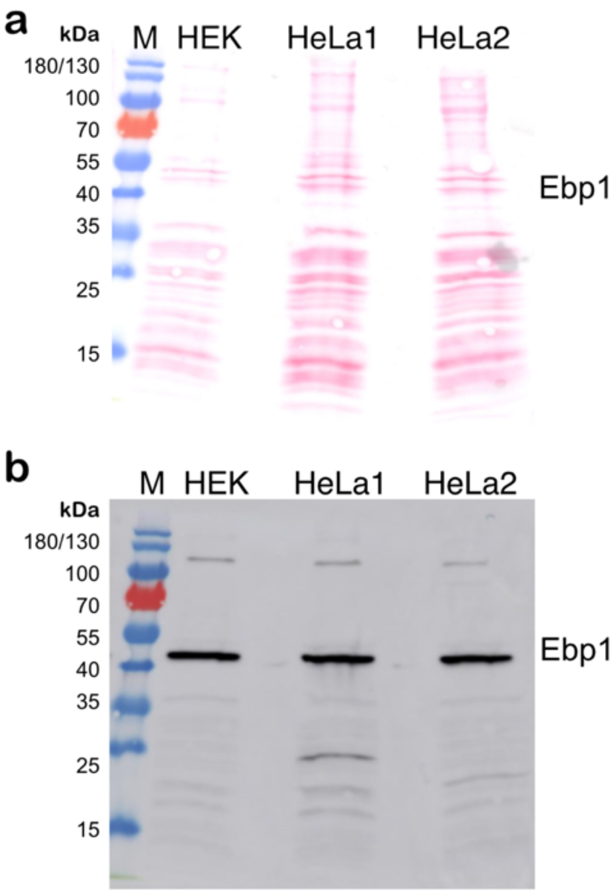

Supplementary Figure 10

Immunoblot analysis of Ebp1-binding to human ribosomes.

a, Ponceau-red stained SDS-PAGE gel of natively purified 80S ribosomes from HEK293 or HeLa (two different preparations) cell cultures. **b**, Ebp1 immunoblot of same gel (Anti-Ebp1 NT antibody, ABE43 Merck KGaA).

Supplementary Table 1

Cryo-EM data collection, refinement and validation statistics

**Data collection and processing**	**Dataset 1**	**Dataset 2**	
Detector	K2	K3	
Nominal Magnification (pre-GIF)	130,000	81,000	
Voltage (kV)	300	300	
Electron exposure (e–/Å^2)	39	37	
Defocus range (μm)	-1.5 – -3.0	-0.5 – -2.0	
Pixel size (Å)	1.07	1.07	
Symmetry imposed	C1	C1	
Initial particle images (no.)	162,886	81,482	
	2-body Ebp1-60S (EMD-10344)	3-body Ebp1-ES27L (EMD-10609)	2-body Ebp1-60S classified ES27L (EMD-10608)
Final particle images (no.)	34,467	34,467	4,864
Map resolution (Å)	3.3	5.7	4.64
FSC threshold	0.143	0.143	0.143
Map resolution range (Å)	3.0 – 15.9	2.9 – 12.8	3.55 – 18.3
	H. sapiens Ebp1-ribosome (PDB-6SXO)		
**Refinement**			
Initial model used (PDB code)	2Q8K, 6EK0		
Model resolution (Å)	3.3		
FSC threshold	0.143		
Model resolution range (Å)	3.3-5.7		
Map sharpening *B* factor (Å^2)	-50 to -200		
Model composition			
Non-hydrogen atoms	14,899		
Protein residues	973		
RNA nucleotides	326		
B factors (Å^2)			
Protein	28.73		
RNA	77.06		
R.m.s. deviations			
Bond lengths (Å)	0.009		
Bond angles (°)	1.349		
Validation			
MolProbity score	1.70		
Clashscore	5.95		
Poor rotamers (%)	1.16		
Ramachandran plot			
Favored (%)	95.32		
Allowed (%)	4.68		
Disallowed (%)	0.00		

Supplementary references

1. Kowalinski, E. et al. The crystal structure of Ebp1 reveals a methionine aminopeptidase fold as binding platform for multiple interactions. *FEBS Lett* **581**, 4450-4 (2007).
2. Li, W. et al. Structural basis for selective stalling of human ribosome nascent chain complexes by a drug-like molecule. *Nat Struct Mol Biol* **26**, 501-509 (2019).
3. Anger, A.M. et al. Structures of the human and Drosophila 80S ribosome. *Nature* **497**, 80-5 (2013).
4. Greber, B.J. et al. Insertion of the Biogenesis Factor Rei1 Probes the Ribosomal Tunnel during 60S Maturation. *Cell* **164**, 91-102 (2016).
5. Natchiar, S.K., Myasnikov, A.G., Kratzat, H., Hazemann, I. & Klaholz, B.P. Visualization of chemical modifications in the human 80S ribosome structure. *Nature* **551**, 472-477 (2017).
