# Supplementary material for: MetAP-like Ebp1 occupies the human ribosomal tunnel exit and recruits flexible rRNA expansion segments
Source: Nat Commun. 2020 Feb 7;11:776. doi: 10.1038/s41467-020-14603-7 (PMC7005732; doi:10.1038/s41467-020-14603-7)
Supplement: Supplementary file 2 — Description of Additional Supplementary Information [file 41467_2020_14603_MOESM2_ESM.pdf]

Description of Additional Supplementary Files

File Name: Supplementary Movie 1
Description: Conformational plasticity of ES27L-B in its exit position. The movie shows a morph between three different cryo-EM reconstructions based on 3D classifications focused on ES27L. The view is as in Fig. 1c top panel rotated 90° counter clockwise.

File Name: Supplementary Movie 2
Description: Conformational plasticity of H59 upon Ebp1-binding. The movie shows a morph between H59 in its locked-position (ribosome without Ebp1, PDB ID 6EK0) and in its docked-position in the Ebp1-ribsome complex. H59 serves as dynamic adaptor with a metazoan-specific bulged-out guanine nucleotide (G2711, red) moving about 10 Å from H53 (locked) to the Ebp1 insert domain (docked). The nucleotides of the H59- closing tetraloop are labelled and shown in colour. View is the same as in Fig. 1c bottom panel.
