# Supplementary material for: MetAP-like Ebp1 occupies the human ribosomal tunnel exit and recruits flexible rRNA expansion segments
Source: Nat Commun. 2020 Feb 7;11:776. doi: 10.1038/s41467-020-14603-7 (PMC7005732; doi:10.1038/s41467-020-14603-7)
Supplement: Supplementary file 5 — Reporting Summary [file 41467_2020_14603_MOESM5_ESM.pdf]

nature research

Corresponding author(s): Stefan Pfeffer and Irmgard Sinning

Last updated by author(s): Jan 15, 2020

Reporting Summary

Nature Research wishes to improve the reproducibility of the work that we publish. This form provides structure for consistency and transparency in reporting. For further information on Nature Research policies, see Authors & Referees and the Editorial Policy Checklist .

Statistics

For all statistical analyses, confirm that the following items are present in the figure legend, table legend, main text, or Methods section.

n/a	Confirmed	
☒	☐	The exact sample size (*n*) for each experimental group/condition, given as a discrete number and unit of measurement
☒	☐	A statement on whether measurements were taken from distinct samples or whether the same sample was measured repeatedly
☒	☐	The statistical test(s) used AND whether they are one- or two-sided<br>*Only common tests should be described solely by name; describe more complex techniques in the Methods section.*
☒	☐	A description of all covariates tested
☒	☐	A description of any assumptions or corrections, such as tests of normality and adjustment for multiple comparisons
☒	☐	A full description of the statistical parameters including central tendency (e.g. means) or other basic estimates (e.g. regression coefficient) AND variation (e.g. standard deviation) or associated estimates of uncertainty (e.g. confidence intervals)
☒	☐	For null hypothesis testing, the test statistic (e.g. *F*, *t*, *r*) with confidence intervals, effect sizes, degrees of freedom and *P* value noted<br>*Give P values as exact values whenever suitable.*
☒	☐	For Bayesian analysis, information on the choice of priors and Markov chain Monte Carlo settings
☒	☐	For hierarchical and complex designs, identification of the appropriate level for tests and full reporting of outcomes
☒	☐	Estimates of effect sizes (e.g. Cohen's *d*, Pearson's *r*), indicating how they were calculated

Our web collection on statistics for biologists contains articles on many of the points above.

Software and code

Policy information about availability of computer code

Data collection	*Provide a description of all commercial, open source and custom code used to collect the data in this study, specifying the version used OR state that no software was used.*
Data analysis	*Provide a description of all commercial, open source and custom code used to analyse the data in this study, specifying the version used OR state that no software was used.*

For manuscripts utilizing custom algorithms or software that are central to the research but not yet described in published literature, software must be made available to editors/reviewers. We strongly encourage code deposition in a community repository (e.g. GitHub). See the Nature Research guidelines for submitting code & software for further information.

Data

Policy information about availability of data

All manuscripts must include a data availability statement. This statement should provide the following information, where applicable:

- Accession codes, unique identifiers, or web links for publicly available datasets
- A list of figures that have associated raw data
- A description of any restrictions on data availability

The following cryo-EM densities for the Ebp1-ribosome complex have been deposited in the EMDataBank: Ebp1-60S segment at full-spatial resolution from 2-body multibody refinement (EMD-10610); Ebp1-ES27L segment from 3-body multibody refinement (EMD-10609); Ebp1-60S segment from 2-body multibody refinement after sorting for ES27L conformation (EMD-10608). The atomic coordinates for Ebp1 and interacting ribosomal components have been deposited in the RCSB with accession ID 6SXO. For visualization of the model in context of the entire human 80S ribosome, we recommend to superpose our atomic coordinates to the structure of the human ribosome solved at 2.9 Å resolution (PDB-ID 6EK0), which is virtually identical except for the Ebp1-interacting region. Other data are available from the corresponding authors upon reasonable request.

Field-specific reporting

Please select the one below that is the best fit for your research. If you are not sure, read the appropriate sections before making your selection.

- [x] Life sciences
- [ ] Behavioural & social sciences
- [ ] Ecological, evolutionary & environmental sciences

For a reference copy of the document with all sections, see nature.com/documents/nr-reporting-summary-flat.pdf

Life sciences study design

All studies must disclose on these points even when the disclosure is negative.

Sample size	n/a
Data exclusions	n/a
Replication	n/a
Randomization	n/a
Blinding	n/a

Reporting for specific materials, systems and methods

We require information from authors about some types of materials, experimental systems and methods used in many studies. Here, indicate whether each material, system or method listed is relevant to your study. If you are not sure if a list item applies to your research, read the appropriate section before selecting a response.

Materials & experimental systems

n/a	Involved in the study	
[ ]	[x]	Antibodies
[ ]	[x]	Eukaryotic cell lines
[x]	[ ]	Palaeontology
[x]	[ ]	Animals and other organisms
[x]	[ ]	Human research participants
[x]	[ ]	Clinical data

Methods

n/a	Involved in the study	
[x]	[ ]	ChIP-seq
[x]	[ ]	Flow cytometry
[x]	[ ]	MRI-based neuroimaging

Antibodies

Antibodies used	human anti-Ebp1: ABE43, Lot # 2445967, Merck Millipore; anti-rabbit IgG antibody: 111-035-144, Jackson ImmunoResearch.
Validation	http://www.merckmillipore.com/DE/de/product/msds/MM_NF-ABE43?Origin=PDP

Eukaryotic cell lines

Policy information about cell lines

Cell line source(s)	HeLa-S3 cells
Authentication	None of the cell lines were authenticated
Mycoplasma contamination	All cell lines have been tested negative.
Commonly misidentified lines (See ICLAC register)	*Name any commonly misidentified cell lines used in the study and provide a rationale for their use.*
